# A Conserved Macrophage-to-Hepatic Stellate Cell PDGF Axis in Human MASH Identified by Multi-Cohort sc/snRNA-Seq Re-Analysis

**DOI:** 10.3390/ijms27177920

**Published:** 2026-09-05

**Authors:** Hao Ling, Peiyu Qiu, Yanzhu Hu

**Affiliations:** 1 Department of Surgery, Klinikum Rechts der Isar, TUM School of Medicine and Health, Technical University of Munich, 81675 Munich, Germany; yanzhu.hu@tum.de; 2 Department of Respiratory Medicine, NUTRIM—Institute for Nutrition and Translational Research in Metabolism, Maastricht University Medical Center, 6229 HX Maastricht, The Netherlands; peiyu.qiu@maastrichtuniversity.nl

**Keywords:** MASH/MASLD, scar-associated macrophages, hepatic stellate cells, PDGF, single-nucleus RNA-sequencing, external validation

## Abstract

Metabolic dysfunction-associated steatohepatitis (MASH) progresses through macrophage activation and hepatic stellate cell (HSC)-driven fibrogenesis. As cell-level single-cell discoveries often suffer from pseudoreplication and fail to replicate across cohorts, in this study we critically re-evaluated human liver single-nucleus RNA-sequencing data using a rigorous per-donor statistical framework to dissociate invariant biological signals from technical artifacts. Macrophage and HSC compartments from dataset GSE202379 were profiled across clinical stages (healthy, metabolic dysfunction-associated steatotic liver disease, MASH). Cell-level statistics were aggregated into per-donor metrics, and continuous phenotypic shifts evaluated via the Jonckheere–Terpstra (JT) trend test. Key findings were tested for cross-cohort reproducibility in two independent cohorts (GSE136103 and GSE244832). A 249-gene scar-associated macrophage (SAMac) program showed reproducible, stage-dependent expansion in the discovery cohort (per-donor Jonckheere–Terpstra trend across the disease-only spectrum, MASLD to advanced MASH, z = 2.23, *p* = 0.026 [primary]; z = 2.74, *p* = 0.006 across all four stages including the healthy baseline) and directional replication in external data. Cell–cell communication analysis replicated the receptor-side topology of a conserved macrophage-to-HSC PDGF axis in both external cohorts and the ligand-driven PDGFC→PDGFRB edge in the external snRNA-seq cohort (GSE244832), with PDGFRB being a high-degree hub within the HSC fibrogenic interaction network. Importantly, a previously published global HSC activation module failed to validate (JT *p* = 0.77); however, its core extracellular matrix and *RUNX1*/2 sub-axis remained robust, whereas the widely claimed AP-1/*JUNB* regulatory driver was not supported at the transcript level in the single-nucleus RNA-seq cohort data. These findings are consistent with an isolation-associated immediate-early gene response in enzymatically dissociated scRNA-seq data, given the flat-to-downward transcript-level trend in cryopreserved-tissue cohorts; transcript-level data cannot, however, exclude protein- or phosphorylation-level AP-1 activity. Targetable downstream components within the validated core architecture were annotated. By implementing per-donor verification, we successfully separated robust pathological features of MASH from platform-dependent artifacts. The validated SAMac program and the macrophage–HSC PDGF axis, together with an associated RUNX subprogram, represent high-confidence, reproducibility-filtered candidate therapeutic nodes that require functional validation. These findings establish a reproducibility-first standard for single-cell translational research, and the validated programs are consistent with emerging models of inter-organ inflammatory circuitry in MASH.

## 1. Introduction

Metabolic dysfunction-associated steatohepatitis (MASH) represents a major global health challenge, serving as an important driver of cirrhosis, liver failure, and hepatocellular carcinoma worldwide [1,2,3]. Liver fibrosis severity correlates strongly with long-term patient mortality and is the critical prognostic determinant in MASH [1,2,4]. Chronically damaged hepatic microenvironments trigger an intricate cellular cascade wherein sustained innate immune (macrophage) activation stimulates hepatic stellate cells (HSCs) to transdifferentiate into extracellular matrix (ECM)-secreting myofibroblasts [5,6]. To identify candidate therapeutic targets that can help halt or reverse MASH progression, it will be essential to dissect robust and reproducible molecular interactions that govern macrophage-to-HSC cross-talk [7].

The rapid development of single-cell and single-nucleus RNA sequencing (sc/snRNA-seq) technologies has significantly expanded understanding of intrahepatic cellular heterogeneity [8]. These approaches have facilitated the identification of specialized cellular subpopulations, including scar-associated macrophages (SAMacs), and nominated a vast array of potential candidate ligand–receptor pairs for anti-fibrotic drug development [9,10]. Related single-cell studies in non-alcoholic steatohepatitis/MASH also identified Trem2^hi^ non-alcoholic steatohepatitis-associated macrophages linked to disease severity, HSC-centered intrahepatic signaling hubs, and expanded macrophage–myofibroblast interaction programs, further supporting the utility of sc/snRNA-seq for therapeutic target discovery [3,11]; however, despite the abundance of candidate pathways identified through computational cell–cell communication workflows, the translational pipeline from in silico target discovery to functional and clinical validation remains challenging, with candidate pathways exhibiting potential reproducibility risks during subsequent evaluation [5,7,9]. Accumulating evidence also frames MASH fibrogenesis as part of systemic inter-organ inflammatory circuits, including the emerging spleen–liver axis [12,13]; however, most single-cell studies remain focused on intrahepatic cellular states, and the cross-cohort reproducibility of candidate downstream hepatic programs has not been systematically assessed.

A significant bottleneck contributing to such translational hurdles is that certain single-cell-derived therapeutic targets may be built on fragile statistical or technical foundations, rendering them potentially cohort-specific [14,15]. Pathologically, MASH exhibits profound patient-level heterogeneity and clinical divergence [2]; however, standard single-cell pipelines frequently treat individual cells as independent observations during differential expression and interactome analyses, whereas cells from the same individual are pseudoreplicates, rather than statistically independent samples [14,16]. This approach introduces pseudoreplication bias, which inflates type I errors and false-positive discoveries and can misread between-subject variation as shared biology [14,16,17]. Benchmarking studies indicate that methods accounting for subjects, including pseudobulk and mixed-effects approaches, outperform naïve cell-level models that ignore replicate structure [14,16]. Traditional dissociation workflows can also distort native transcriptional states, and cell-isolation protocols can compromise the accuracy of captured single-cell transcriptome data [8]. Consequently, without rigorous cross-cohort validation, adequately powered replication, and a reproducibility-focused analytical boundary, the nomination of pharmacological targets based on single-cell analyses remains vulnerable to cohort-specific or platform-dependent conclusions [9,15,18].

To address this concern, we conducted a systematic re-evaluation of a detailed human MASH liver dataset (GSE202379), which captures a continuous disease spectrum from steatotic liver to advanced MASH. To ensure our discoveries reflect genuine clinical progression, we implemented a per-donor aggregation framework and applied the Jonckheere–Terpstra trend test to donor-level summaries across ordered disease stages, thereby reducing pseudoreplication risk and testing stage-associated monotonic trends at the donor level [14,19,20]. Importantly, we conducted cross-cohort (external) validation of the structural and transcriptomic robustness of the core fibrogenic components identified using two independent external cohorts generated with distinct isolation methodologies (GSE136103 and GSE244832). Through this multi-cohort validation approach, we sought to critically evaluate which regulatory axes genuinely drive human MASH fibrosis and eliminate results reflecting cell stress induced by technical processes used during RNA isolation. Our findings refine understanding of the MASH microenvironment and we describe a robust, cross-cohort-validated molecular framework to inform precise therapeutic intervention.

## 2. Results

### 2.1. A Per-Donor, Externally Validated Re-Analysis of Macrophage–HSC Biology in MASH

**Figure 1:**
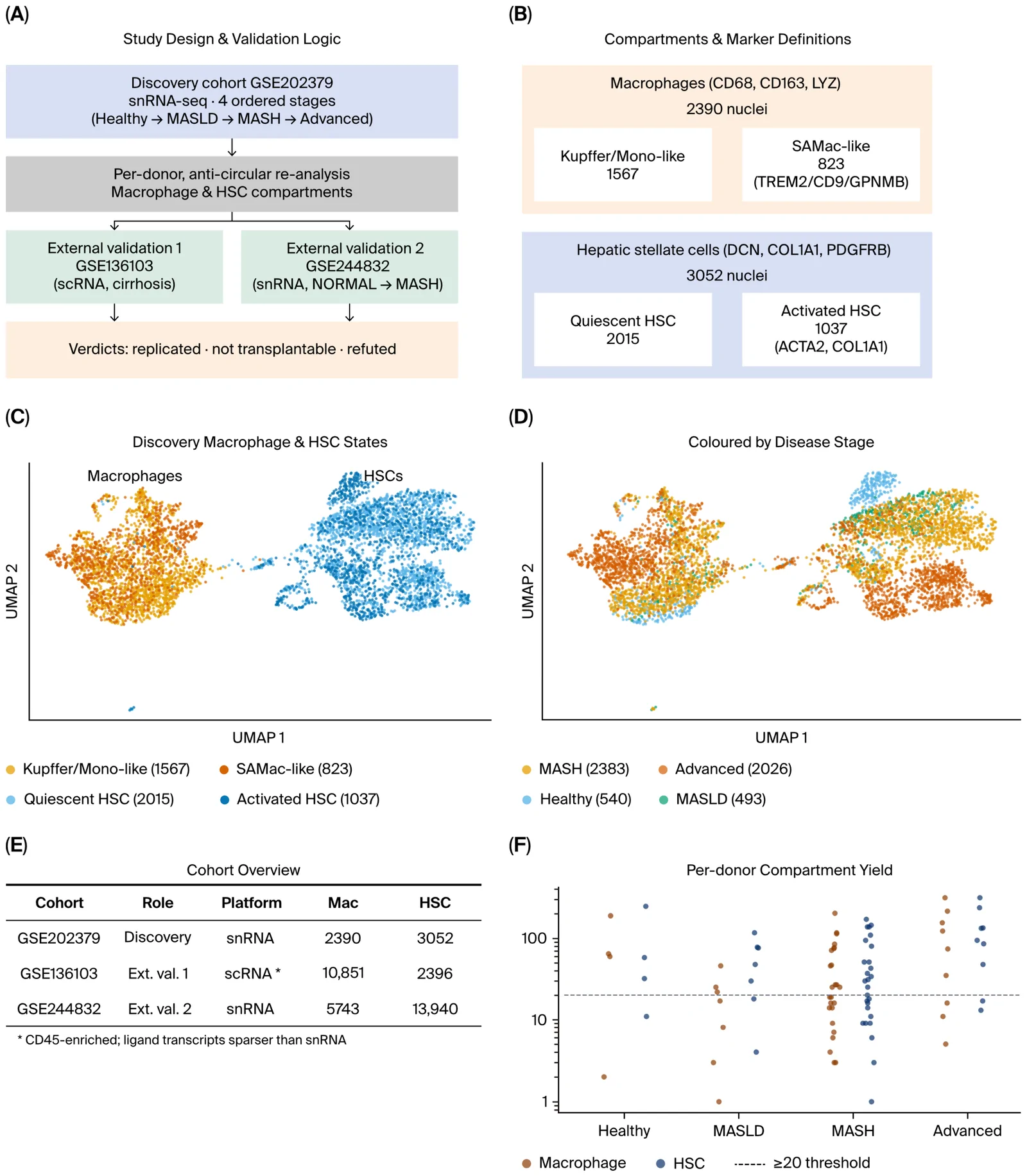
A metabolic dysfunction-associated steatohepatitis (MASH) snRNA-seq atlas generated by rigorous reanalysis and independent external validation of macrophage-hepatic stellate cell (HSC) crosstalk. (**A**) Study design: macrophage and HSC compartments were defined using the discovery snRNA-seq dataset (GSE202379) and tested in two external cohorts (GSE136103, GSE244832). (**B**) Compartment definitions, nuclei counts, and markers. Joint uniform manifold approximation and projection (UMAP) of 5442 discovery macrophage and HSC nuclei colored by (**C**) cell state and (**D**) disease stage (computed for visualization only; all quantitative results derive from per-donor analyses). (**E**) Cohort overview, roles, platforms, and per-compartment cell numbers. (**F**) Per-donor nuclei yield per compartment across clinical stages. * GSE136103: scRNA-seq of CD45+ and CD45− NPCs (immune-biased recovery; see Methods).

We re-analyzed the GSE202379 snRNA-seq human liver atlas across the MASLD-to-MASH spectrum; the macrophage (2390 nuclei: 1567 Kupffer/Mono-like, 823 SAMac-like) and HSC (3052 nuclei: 2015 quiescent, 1037 activated) compartments were defined using canonical markers (Figure 1A,B), and the joint UMAP embedding is shown colored by cell state and disease stage (Figure 1C,D). To avoid the pseudoreplication and circularity that commonly inflate claims based on single-cell data, all stage trends were tested per donor and all external comparisons used signature-independent metrics. Three resulting discoveries, including SAMac stage-expansion, a macrophage → HSC PDGF axis, and a global HSC activation module, were subsequently tested in data from two independent cohorts: GSE136103 (scRNA-seq) and GSE244832 (snRNA-seq) (Figure 1A,E; Table 1). Validation outcomes for these three findings are summarized in Table 2 (expanded per-test cross-cohort verdict matrix in Table S22). Per-donor nuclei yields per compartment across clinical stages are shown in Figure 1F, with per-donor macrophage and HSC nuclei counts and donor-feasibility summaries provided in Table S5. Companion bulk-tissue and T-cell compartment analyses are provided for reference: genome-wide bulk RNA-seq differential expression with post-ComBat batch-correction quality control (Table S1), MCP-counter immune/stromal population deconvolution (Table S2), T-cell compartment pseudobulk differential expression (Table S3), and hub-gene differential expression cross-validated in an augmented bulk cohort (Table S4).

### 2.2. SAMacs Expressed a Coherent Intrinsic Program That Expands with Disease Stage

**Figure 2:**
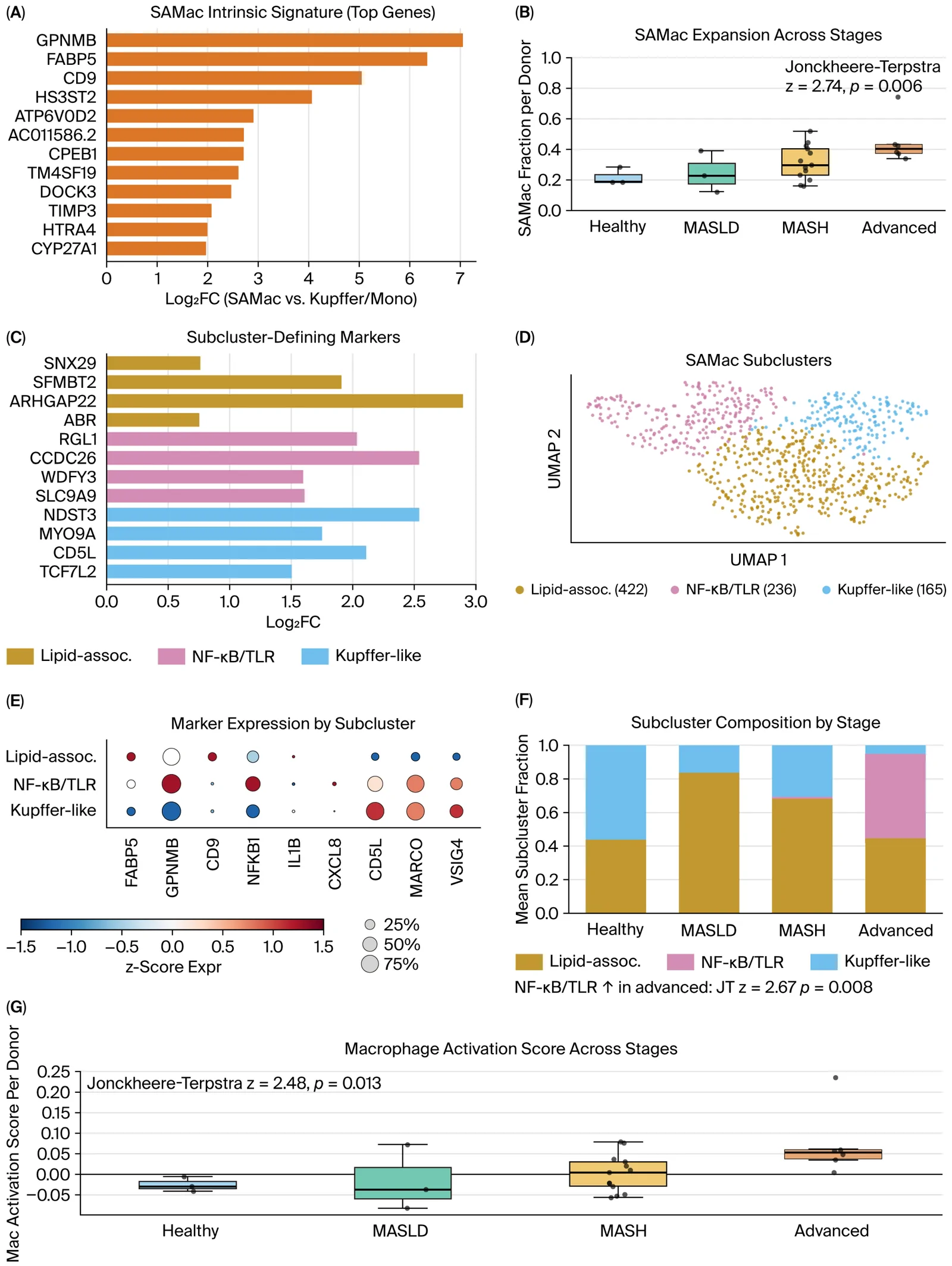
Scar-associated macrophages (SAMacs) express a coherent intrinsic program that expands with MASH stage (GSE202379). (**A**) Top genes of the 249-gene SAMac intrinsic signature according to log2 fold-change. (**B**) Per-donor SAMac fraction increased across stages; the primary disease-only trend (MASLD to advanced MASH) was Jonckheere–Terpstra (JT) z = 2.23, *p* = 0.026, and JT z = 2.74, *p* = 0.006 across all four stages including the healthy baseline shown (Table S23). (**C**) Top markers of three reproducible SAMac substates. (**D**) SAMac subcluster uniform manifold approximation and projection (UMAP) (823 nuclei; lipid-associated, NF-κB/TLR-activated, Kupffer-like). (**E**) Substate marker dot plot (z-scored mean expression, dot size = percent expressing). (**F**) Substate composition by stage (NF-κB/TLR-activated rose in the advanced group, JT z = 2.67, *p* = 0.008). (**G**) Per-donor macrophage activation score increased across stages (disease-only JT z = 2.10, *p* = 0.036; four-stage JT z = 2.48, *p* = 0.013). The upward arrow (↑) in (**F**) denotes a significant increase in the NF-κB/TLR-activated substate fraction in the advanced-stage group.

Analysis of discovery cohort data (GSE202379) demonstrated that the SAMac-like state was defined by a 249-gene intrinsic signature (edgeR, FDR < 0.05; Table S8), with expression of the genes *GPNMB* (log2FC + 7.05), *FABP5* (+6.35), *CD9* (+5.05), and *HS3ST2* (+4.07) being most strongly altered, consistent with the conserved lipid- and scar-associated macrophage phenotype (Figure 2A) [6]. Because the discovery healthy baseline comprised only two usable donors (Section 4.2), the disease-only spectrum (MASLD → MASH → advanced MASH) was pre-specified as the primary progression analysis. Across this disease-only spectrum the per-donor SAMac fraction increased monotonically (JT z = 2.23, *p* = 0.026; median 22.7% in MASLD to 40.3% in advanced MASH), and the per-donor macrophage activation score increased in parallel (JT z = 2.10, *p* = 0.036) (Figure 2B,G; stage-trend statistics in Table S6 and full per-stage descriptives in Table S10). The trend was directionally unchanged when the healthy baseline was included as a four-stage sensitivity check (HL2 retained; fraction JT z = 2.74, *p* = 0.006; score JT z = 2.48, *p* = 0.013) and when the healthy-baseline outlier HL2 was removed (fraction z = 2.50, *p* = 0.012; score z = 2.34, *p* = 0.020), confirming that the stage-dependent increase was not an artifact of the healthy anchor (full sensitivity grid in Table S23; per-analysis donor counts in Table S24). Sub-clustering analysis resolved three reproducible SAMac substates—Kupffer-like, lipid-associated, and NF-κB/TLR-activated—each with distinct markers (Table S15), and the NF-κB/TLR-activated substate also expanded with clinical stage (JT z = 2.67, *p* = 0.008; substate-level analysis restricted to donors with ≥10 SAMac nuclei, *n* = 18; Table S24) (Figure 2C–F). Changes in the canonical inflammatory markers, *TREM2*, *SPP1*, and *OLR1*, did not reach intrinsic significance, indicating that the SAMac program is defined by a broader lipid-handling and ECM-interacting module, rather than by these markers alone. An exploratory diffusion-pseudotime ordering of the three SAMac substates arranged them along a Kupffer-like → lipid-associated → NF-κB/TLR-activated axis (JT z = 17.0, *p* < 1 × 10^−60^). Further, *PDGFC* expression followed a U-shaped pattern, with a relative trough in the lipid-associated substate (Kruskal–Wallis *p* = 2.9 × 10^−15^; Supplemental Figure S1); this pattern was preserved in donor random-intercept mixed model analysis (Supplemental Figure S3).

### 2.3. A Macrophage-to-HSC PDGF Crosstalk Axis and an Associated RUNX Subprogram Converge on PDGFRB as a Network Hub

**Figure 3:**
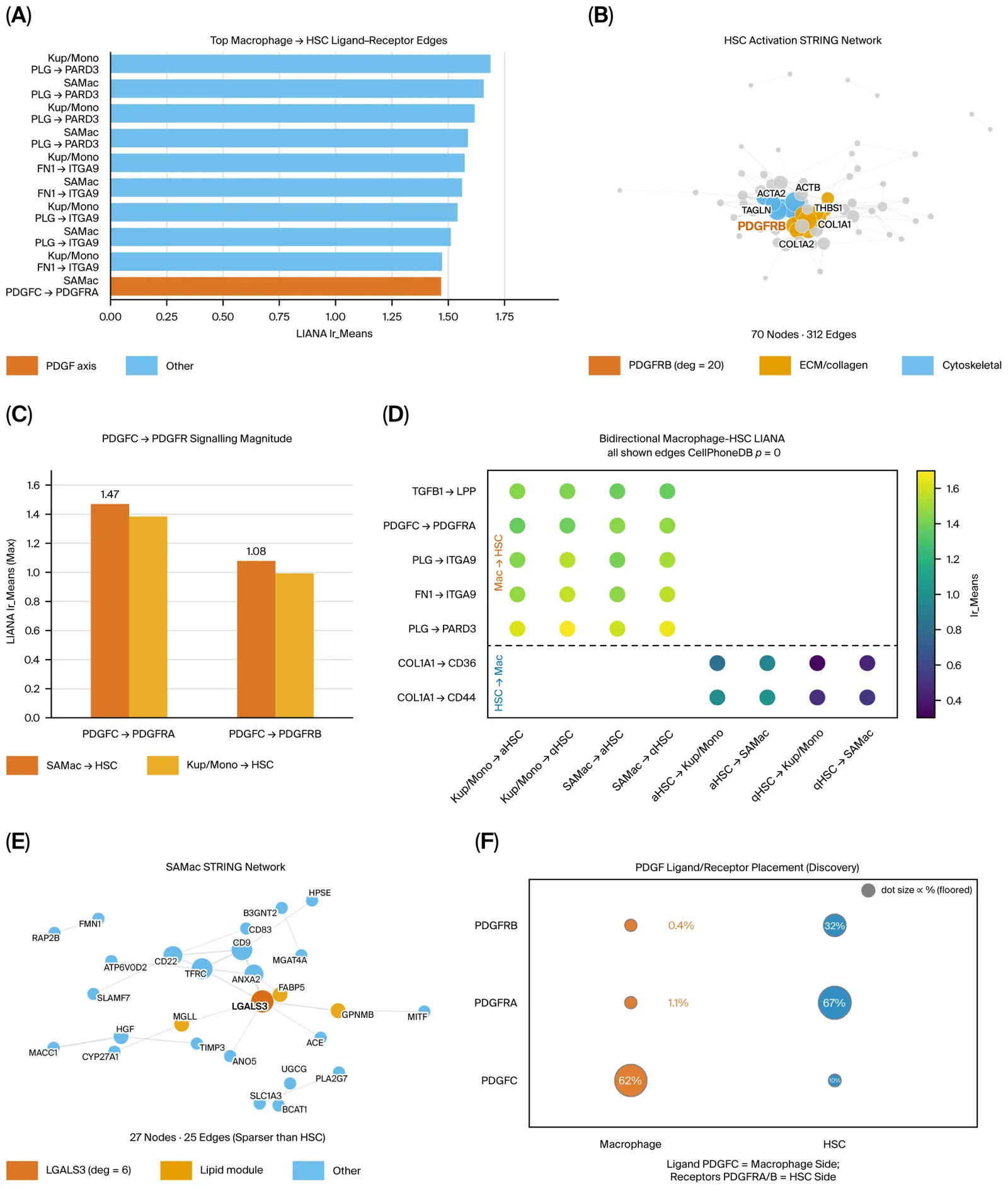
A macrophage-to-hepatic stellate cell (HSC) PDGF crosstalk axis, with an associated RUNX subprogram, converges on PDGFRB as a fibrogenic network hub (GSE202379). (**A**) Top macrophage → HSC LIANA ligand–receptor edges; PDGF axis highlighted (orange). (**B**) HSC activation STRING network (70 nodes, 312 edges). PDGFRB (red) is a degree-20 hub among extracellular matrix/collagen and cytoskeletal nodes. (**C**) LIANA magnitude scores for macrophage-sourced PDGFC → PDGFRA and PDGFC → PDGFRB edges. (**D**) Bidirectional macrophage–HSC dot plot generated using LIANA: top macrophage → HSC edges and reciprocal HSC → macrophage collagen-sensing edges (COL1A1 → CD44/CD36) (color = magnitude of ligand–receptor (lr) mean expression value; all displayed edges CellPhoneDB *p* < 0.001, i.e., below the 1000-permutation detection limit). (**E**) Scar-associated macrophage (SAMac) STRING network (27 nodes, 25 edges) with LGALS3 hub. (**F**) PDGF placement (discovery): *PDGFC* marks macrophages, *PDGFRA*/B mark HSCs. In (**B**,**E**), node size is proportional to the protein’s interaction degree in the STRING network; in (**F**), dot size is proportional to the percentage of cells expressing the gene (floored). Arrows (→) denote the signaling direction from the ligand-expressing source cell type to the receptor-expressing target cell type.

Modeling using the LIANA framework recovered a donor-pervasive, bidirectional macrophage–HSC circuit from the discovery cohort data (GSE202379) (Figure 3A,D). The dominant macrophage → HSC edges were PDGFC → PDGFRA and PDGFC → PDGFRB (CellPhoneDB permutation *p* < 0.001; the strongest of these edges reached a LIANA magnitude rank-aggregate score of 5.0 × 10^−5^), with macrophages as the ligand source and HSCs as the receptor compartment (Figure 3A,C,D). Reciprocally, activated HSCs were found to signal to macrophages through collagen sensing (COL1A1 → CD44/CD36; CellPhoneDB *p* < 0.001) (Figure 3D). PDGFRB was a high-degree hub (degree 20), embedded among core fibrogenic nodes, including COL1A1 (28), TAGLN (28), THBS1 (26), ACTA2 (25), and CCN2/CTGF (22) in an HSC activation protein–protein interaction network (Figure 3B; Table S9). The SAMac network was sparser and centered on LGALS3 (Figure 3E), with compartment-resolved expression analysis placing *PDGFC* in macrophages and *PDGFRA*/*PDGFRB* on HSCs in the discovery cohort (Figure 3F). Together these analyses nominate a macrophage → HSC PDGF axis, anchored on PDGFRB, as a candidate central fibrogenic circuit in the discovery cohort, with an associated RUNX transcriptional subprogram in HSCs. The PDGF pathway and the RUNX subprogram were supported by separate lines of evidence (network topology and donor-level receptor coupling for PDGF; differential expression for RUNX); a direct PDGF → RUNX regulatory link is hypothesized and remains to be tested functionally.

**Figure 4:**
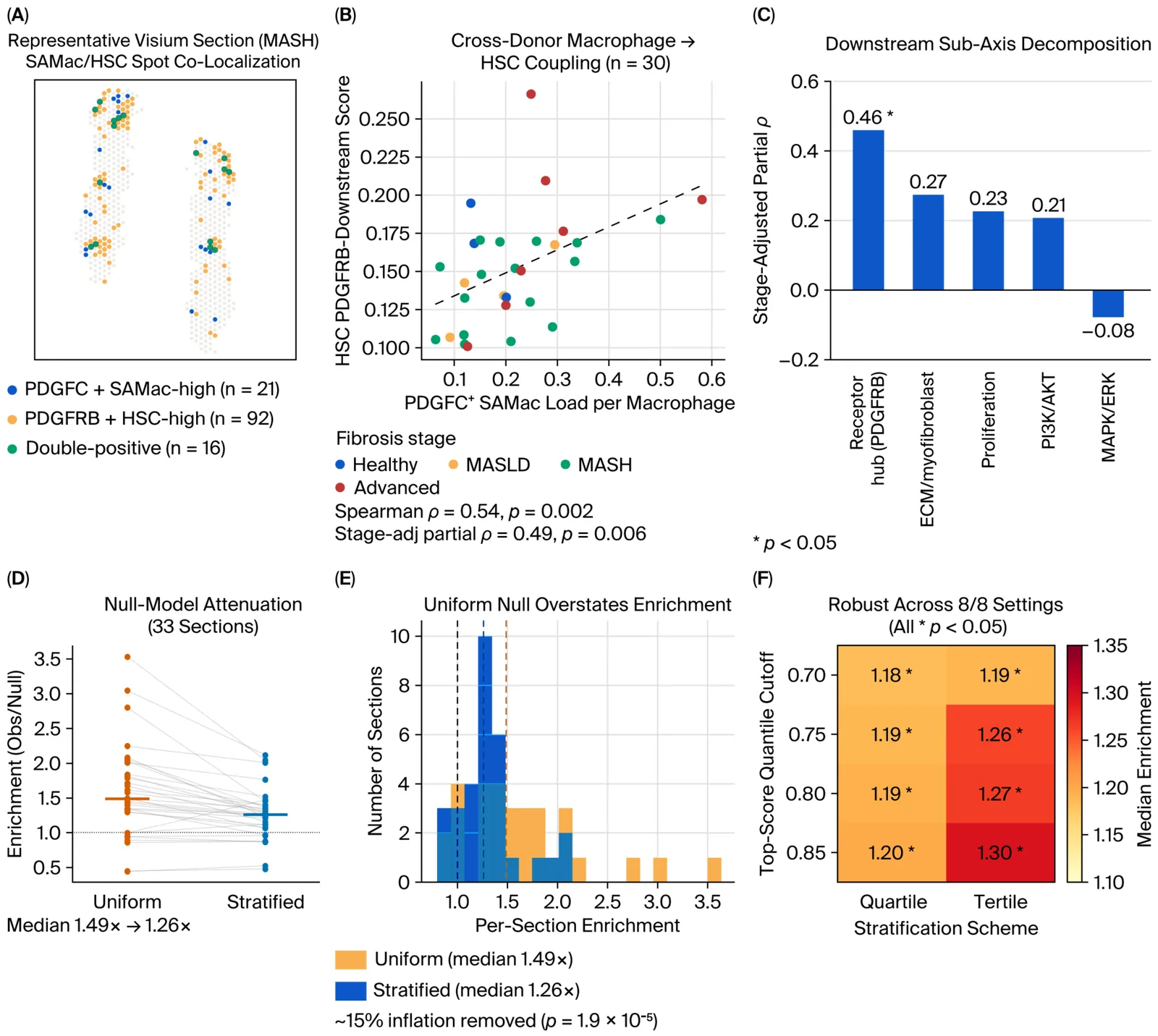
Donor-level and spatial validation of the macrophage → hepatic stellate cell (HSC) PDGF axis. (**A**) A representative MASH Visium section showing spatial co-localization of *PDGFC*^+^ scar-associated macrophage (SAMac)-high spots, *PDGFRB*^+^ HSC-high spots, and double-positive spots. (**B**) Across donors (*n* = 30, GSE202379), the per-donor *PDGFC*^+^ SAMac load was correlated with the HSC PDGFRB-downstream score (Spearman ρ = 0.54, *p* = 0.002; stage-adjusted partial ρ = 0.49, *p* = 0.006); spot color indicates fibrosis stage. (**C**) Decomposition of the HSC downstream response into sub-axes: only the PDGFRB receptor hub was significantly coupled after stage adjustment (partial ρ = 0.46, *p* = 0.011; other sub-axes n.s.). (**D**) Spatial neighbor-enrichment attenuates from a uniform null (median 1.49×) to a stratified null that shuffles HSC-high labels within HSC-score tertiles (median 1.26×; *p* = 1.7 × 10^−4^, 33 sections). (**E**) The uniform null overstates enrichment by approximately 15% (paired Wilcoxon *p* = 1.9 × 10^−5^); the residual enrichment (approximately 1.26×) reflects genuine focal co-localization. (**F**) Stratified-null median enrichment remained significant across all eight quantile × stratification settings (1.18–1.30×, all *p* < 0.05). Panel (**A**) is illustrative of a single section; the inferential enrichment estimate is the stratified value (median 1.26×), not the uncorrected single-section maximum. Arrows (→) in (**B**,**D**) denote the macrophage-to-HSC direction and the change from the uniform to the stratified null median, respectively. The dashed line in (**B**) shows the linear trend across donors; in (**E**), vertical dashed lines mark the null expectation (enrichment = 1.0, black) and the median of each null distribution (uniform, orange; stratified, blue); the dotted horizontal line in (**D**) marks the null expectation (enrichment = 1.0).

To move beyond a qualitative description of this circuit, we quantified its coordination at the level of individual donors (Figure 4B,C). Across donors (*n* = 30 donors with both a qualifying HSC compartment [≥20 nuclei] and macrophage data for the *PDGFC*+ SAMac abundance; Table S24), the per-donor abundance of *PDGFC*^+^ SAMac cells was positively correlated with the HSC PDGFRB-downstream transcriptional response (Spearman ρ = 0.54, *p* = 0.002), and this relationship persisted after adjustment for fibrosis stage (stage-adjusted partial ρ = 0.49, *p* = 0.006), indicating that donor-to-donor variation in the macrophage ligand source tracked HSC receptor-pathway activation independently of disease severity. This stage-adjusted association was robust to a finer five-level ordinal stage recoding, which left the partial correlation essentially unchanged (partial ρ = 0.49 under both the coarse four-level and the finer five-level stage codings, which were themselves near-perfectly concordant: Spearman ρ = 0.995, *p* = 5 × 10^−30^) (Supplemental Figure S4). Donor-level macrophage–HSC coupling was stable across per-donor cell-count thresholds (partial ρ = 0.50, 0.49 and 0.42 at ≥10, ≥20, and ≥30 HSCs per donor). On decomposition of the HSC downstream response into functional sub-axes, only the PDGFRB receptor-hub sub-axis remained significantly coupled after stage adjustment (partial ρ = 0.46, *p* = 0.011), whereas the ECM/myofibroblast, proliferation, PI3K/AKT, and MAPK/ERK sub-axes did not, localizing the cross-donor coupling to the receptor hub itself. As a complementary ordinal-association analysis, leave-one-out machine-learning models relating per-donor macrophage and HSC compartment features to ordinal disease stage are reported in Table S11.

### 2.4. External Validation I: SAMac Stage-Expansion Replicates Across Cohorts

**Figure 5:**
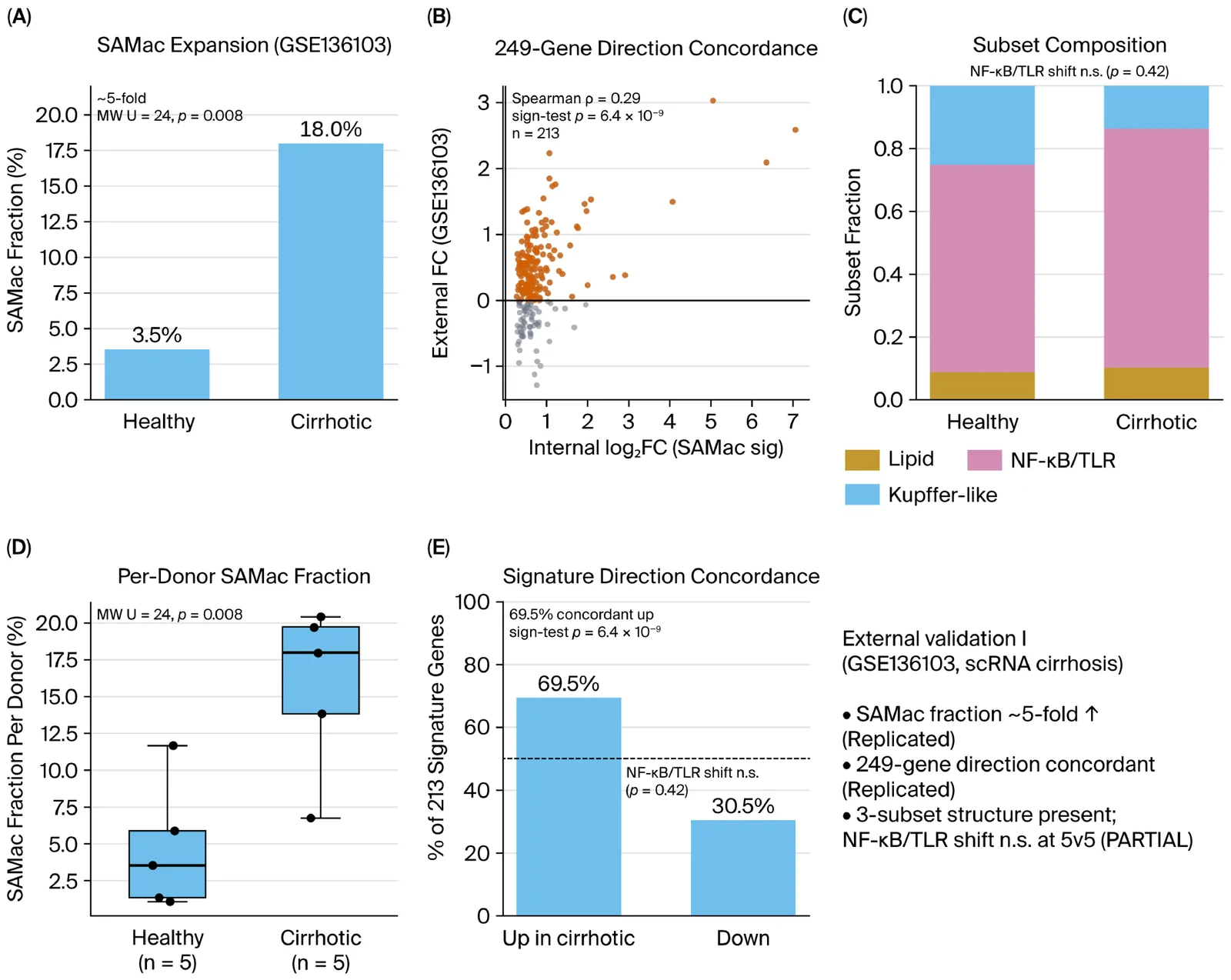
External validation I: replication of scar-associated macrophage (SAMac) stage-expansion (GSE136103). (**A**) The SAMac fraction was higher in cirrhotic than healthy liver (3.5% of macrophages in healthy vs. 18.0% in cirrhotic; the pooled count 1009/10,851 macrophages = 9.3% is the overall SAMac fraction across all samples, not either single condition; per-donor Mann–Whitney *p* = 0.008, *n* = 5 healthy vs. 5 cirrhotic, panel (**D**); *TREM2*/*CD9*/*GPNMB* anchor). (**B**) Direction concordance of the 249-gene SAMac signature between discovery and validation per-donor log2 fold-changes (Spearman rho = 0.29; sign test *p* = 6.4 × 10^−9^; *n* = 213; anchors excluded). (**C**) SAMac three-substate composition, healthy versus cirrhotic (NF-κB/TLR shift not significant, *p* = 0.42). (**D**) Per-donor SAMac fraction, healthy versus cirrhotic (*n* = 5 each; Mann–Whitney U = 24, *p* = 0.008). (**E**) Of 213 signature genes, 69.5% were concordantly higher in cirrhosis versus healthy samples. In (**B**), each dot represents one signature gene (orange, external fold-change > 0, i.e., concordantly up-regulated in GSE136103; grey, external fold-change < 0), and the solid horizontal line marks no external change (fold-change = 0); the dashed horizontal line in (**E**) marks the 50% chance level. ‘↑’ denotes fold increase; ‘n.s.’, not significant.

In the GSE136103 dataset, analysis of the signature-independent *TREM2*/*CD9*/*GPNMB* anchor indicated that the SAMac fraction rose from 3.5% of macrophages in healthy liver to 18.0% in cirrhotic liver (per-condition fractions); the count of 1009 of 10,851 macrophages refers instead to the overall SAMac fraction pooled across all samples (9.3%), not to either single condition, and the reported significance is the per-donor test (Mann–Whitney *p* = 0.008; *n* = 5 healthy vs. 5 cirrhotic; Figure 5A,D). Analysis using the 249-gene SAMac signature also reproduced at the level of direction; with canonical anchors excluded, per-donor pseudobulk log2 fold-changes were positively concordant between the discovery and validation datasets (Spearman rho = 0.29; up-fraction 69.5%; sign test *p* = 6.4 × 10^−9^) (Figure 5B,E), and the per-donor SAMac fraction was higher in cirrhotic than healthy liver (Mann–Whitney U = 24, *p* = 0.008; Figure 5D). The three SAMac substates were also recovered, with a non-significant shift toward the NF-κB/TLR-activated subset in the cirrhosis group (66% to 76%; *p* = 0.42) (Figure 5C). Hence, SAMac stage-expansion is a robust, replicable feature of human liver disease (per-cohort validation summary in Table S20).

### 2.5. External Validation II: The HSC Activation Module Was Not Transplantable; An ECM/RUNX Sub-Axis Replicated and an AP-1 Driver Was Not Supported at the Transcript Level

**Figure 6:**
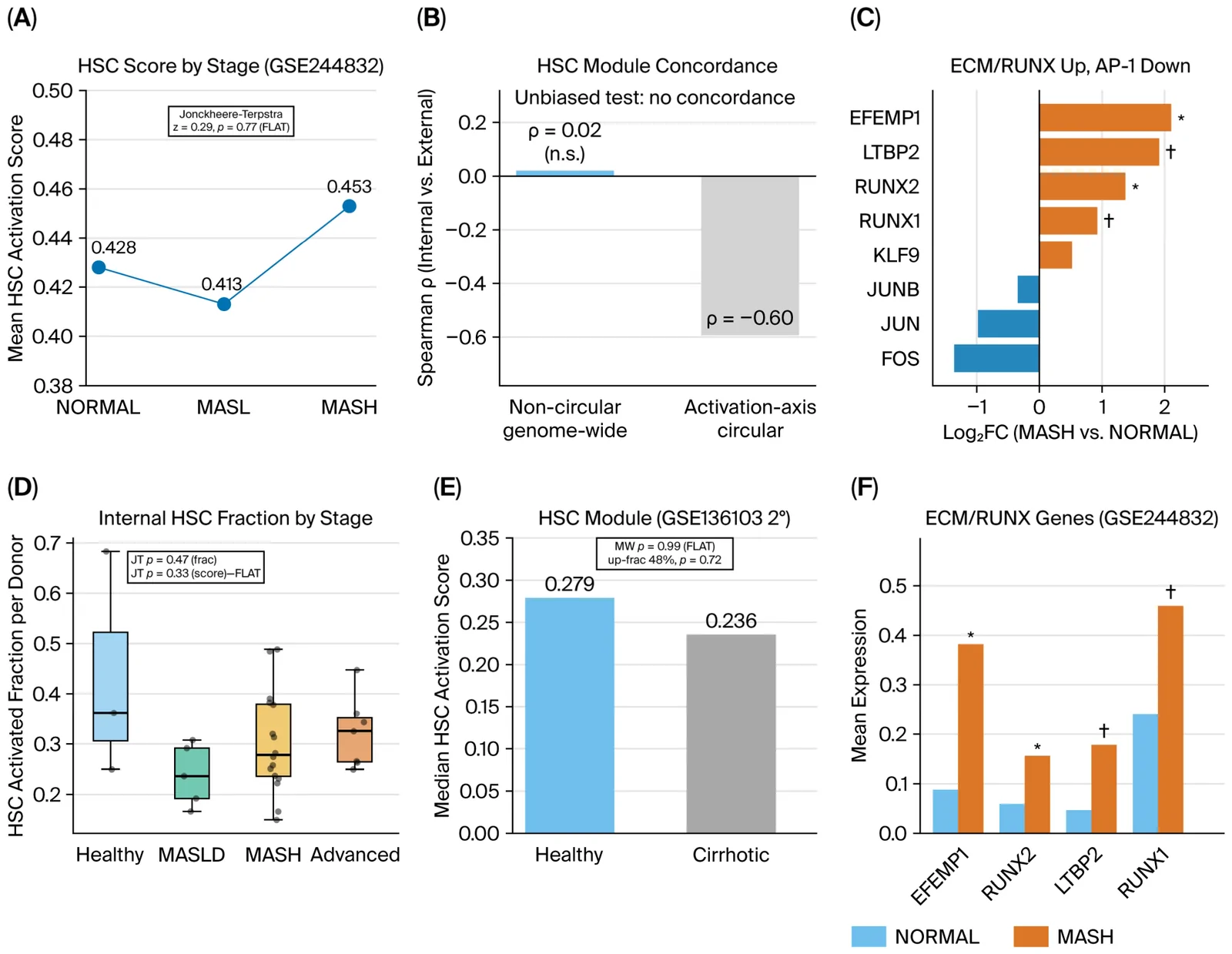
External validation II: the hepatic stellate cell (HSC) activation module was not transplantable; an ECM/RUNX sub-axis replicated, and the AP-1 driver was not supported at the transcript level (GSE244832). (**A**) Median HSC activation score was flat across the NORMAL, MASL, and MASH groups (Jonckheere–Terpstra *p* = 0.77). (**B**) Unbiased genome-wide direction concordance was absent (Spearman rho = 0.02; i.e., no concordance, not a weak positive correlation). (**C**) Per-gene log2 fold-changes (MASH vs. NORMAL): ECM/RUNX genes (*EFEMP1*, *LTBP2*, *RUNX1*/2) up with significant FDR (markers as defined in the key below), AP-1 family flat-to-down. (**D**) Internal activated-HSC fraction was also flat across stages in the discovery cohort (GSE202379; four-stage JT *p* = 0.47 with HL2 retained, as plotted, and *p* = 0.28 with HL2 removed; disease-only JT *p* = 0.11; all non-significant). (**E**) The HSC module was flat in the GSE136103 dataset (Mann–Whitney *p* = 0.99). (**F**) ECM/RUNX gene expression, MASH vs. NORMAL (same significance markers as in (**C**)). Significance markers in (**C**,**F**): * FDR < 0.05; † FDR < 0.1. ‘n.s.’, not significant.

In contrast to the macrophage program, validation analyses indicated that the global HSC activation signature (Table S8) did not generalize. In GSE244832 data, HSC activation score did not increase across the NORMAL, MASL, and MASH groups (median = 0.428, 0.413, and 0.453, respectively; JT *p* = 0.77; Kruskal–Wallis *p* = 0.69) (Figure 6A; Table S19) and genome-wide direction concordance was essentially absent between the discovery and validation datasets (Spearman rho = 0.02; up-fraction 50%; sign test n.s.) (Figure 6B). Likewise, internal activated HSC fraction was unchanged across clinical stages in either the GSE202379 (four-stage JT *p* = 0.47 with HL2 retained and *p* = 0.28 with HL2 removed) (Figure 6D) or GSE136103 cohorts (Mann–Whitney *p* = 0.99) (Figure 6E). An earlier concordance estimate that re-used the activation signature both to define the HSC activation axis and as the variable being correlated (self-correlation rho = −0.60) was rejected as circular: because the same signature genes define both sides of the comparison, this statistic is a form of double-dipping and does not test external replication. It was therefore replaced by the unbiased, signature-independent genome-wide MASH-vs-NORMAL direction concordance reported above (rho = 0.02). Importantly, this null finding was specific to the HSC activation module as a whole: a focused ECM/RUNX sub-axis replicated, with *EFEMP1* (log2FC + 2.1), *LTBP2* (+1.9), *RUNX2* (+1.4), and *RUNX1* (+0.9) significantly or near-significantly upregulated in MASH (*EFEMP1* and *RUNX2* FDR = 0.019) (Figure 6C), consistent with the original GSE244832 report [21] (Figure 6F). Conversely, the previously proposed AP-1/*JUNB* regulatory driver was not supported at the transcript level: levels of the entire AP-1 family (*JUN*, *JUNB*, *FOS*, *FOSB*, *JUND*, *ATF3*, *FOSL2*) were flat-to-down and *KLF9* showed a non-significant positive shift (log2FC + 0.52), with none of these genes reaching significance in MASH (all *p* ≥ 0.18) (Figure 6C), consistent with the discovery-cohort AP-1 signal reflecting a dissociation-associated immediate-early gene response in enzymatic scRNA-seq rather than a stage-dependent disease signal; because snRNA-seq under-represents cytoplasmic immediate-early transcripts, this transcript-level absence does not by itself exclude protein- or phosphorylation-level AP-1 activity, and may also reflect differences in RNA capture, cohort composition, or disease stage between the enzymatically dissociated scRNA-seq and snRNA-seq datasets rather than a proven technical artifact. In an exploratory decomposition of this module into functional submodules, we found that fibrillar-ECM and growth-factor-signaling sub-signatures showed nominal stage-associated replication in the external cohort (Spearman ρ = 0.53 and 0.47; nominal *p* = 0.022 and 0.042, respectively), although neither survived multiple-testing correction across submodules (best FDR approximately 0.10); hence, these submodule trends are considered exploratory only (Supplemental Figure S2). An exploratory stage-stratified analysis of the HSC compartment—early- and late-stage pseudobulk differential expression, a curated senescence-marker panel, pathway enrichment of late-stage genes, and per-donor module scoring—is reported in Table S17.

### 2.6. External Validation III: Macrophage-to-HSC PDGF Topology Was Conserved and the LIANA Edge Replicated in snRNA-Seq Data

**Figure 7:**
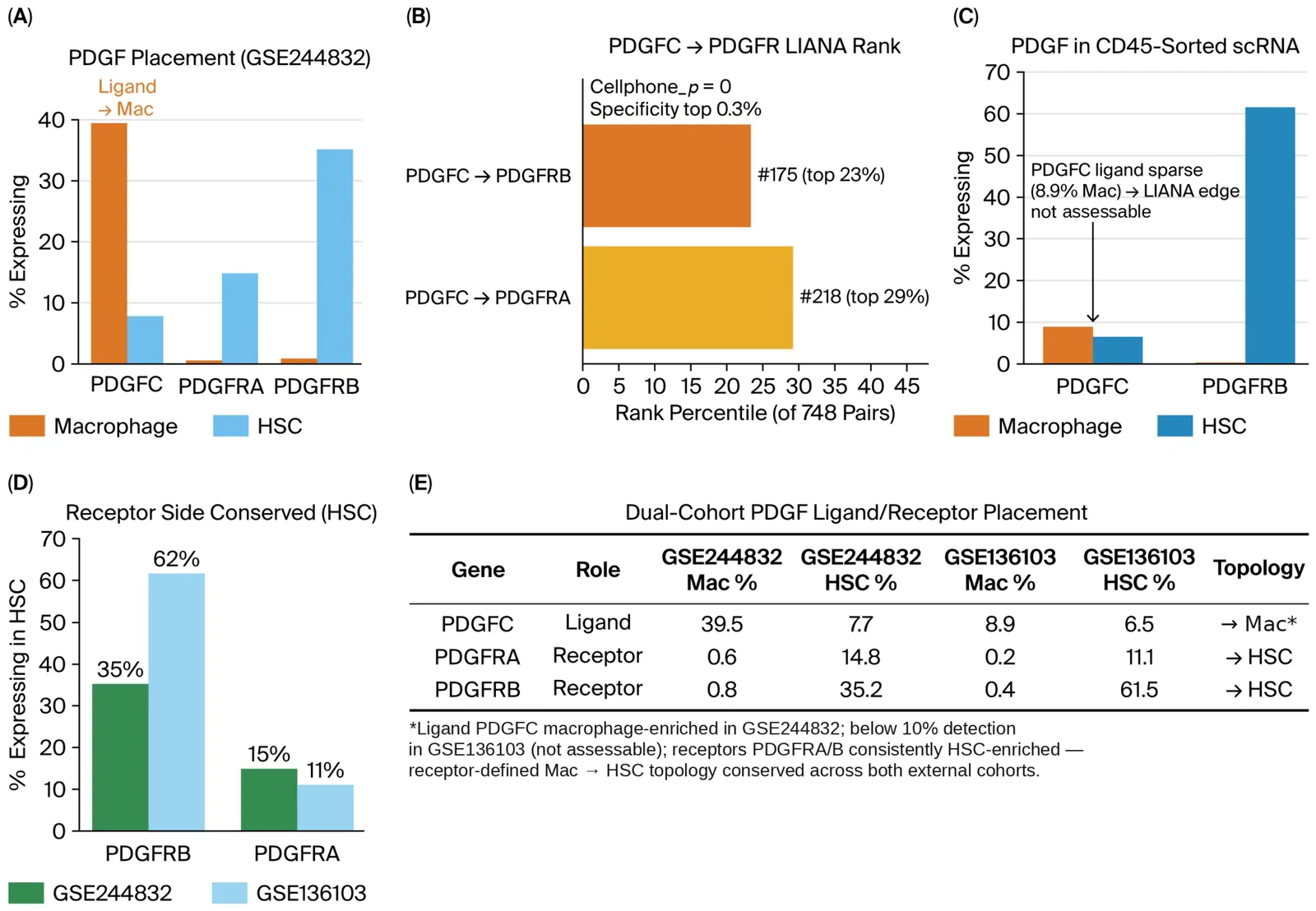
External validation III: macrophage-to-hepatic stellate cell (HSC) PDGF topology is conserved and the LIANA edge replicates in snRNA-seq data. (**A**) PDGF localization in GSE244832: PDGFC (ligand) was enriched in macrophages, while receptors (PDGFRB/PDGFRA) were enriched in HSCs (percent expressing). (**B**) LIANA macrophage → HSC PDGF edges were significant and specific in GSE244832 data (CellPhoneDB *p* < 0.001; PDGFC → PDGFRB rank 175/748 by magnitude). (**C**) Cross-platform caveat in GSE136103: *PDGFC* fell below the 10% expression-proportion detection threshold in CD45-sorted scRNA-seq data, so the ligand-driven edge could not be assessed (‘not assessable’, Table 2). (**D**) The receptor side is conserved, with *PDGFRB* enriched in HSCs in both cohorts (61.5% and 35.2%). (**E**) Dual-cohort localization summary, confirming the directional macrophage → HSC topology. Arrows (→) denote the signaling direction from ligand-expressing macrophages to receptor-expressing HSCs (in (**C**), ‘→’ denotes ‘leads to’); ‘#’ denotes the magnitude rank of an edge among all 748 tested ligand–receptor edges (e.g., #175 = rank 175 of 748).

We found that PDGF crosstalk topology was conserved across cohorts. In both validation datasets, the PDGF receptors—PDGFRB and PDGFRA—were enriched on HSCs (GSE244832 *PDGFRB* 35.2% of HSCs vs. 0.8% of macrophages; GSE136103 *PDGFRB* 61.5% vs. 0.4%), while the ligand, PDGFC, was enriched in macrophages in the snRNA-seq cohort (GSE244832 39.5% of macrophages vs. 7.7% of HSCs) (Figure 7A; Table S21). LIANA modeling using GSE244832 recovered the macrophage → HSC PDGFC → PDGFRB edge as significant and highly cell-type-specific but of only moderate interaction magnitude (CellPhoneDB permutation *p* < 0.001; LIANA RobustRankAggregate consensus scores, interpretable as *p*-values rather than percentiles: specificity rank-aggregate = 0.003, magnitude rank-aggregate = 0.40; ordering the 748 tested edges by this magnitude score places the edge 175th, ~top 23%) (Figure 7B; Table S21). The receptor side of the axis was also preserved on analysis of the GSE136103 dataset (PDGFRB the dominant HSC receptor), whereas the ligand-driven edge could not be assessed, because *PDGFC* fell below the 10% expression-proportion (expr_prop) detection threshold used for the external cohorts and was captured in only 8.9% of macrophages in CD45-sorted scRNA-seq (Figure 7C); we interpret this as a ligand-specific platform/sorting limitation, not a refutation of the axis, as the receptor side was conserved in both cohorts (*PDGFRB* HSC-enriched: 35.2% in GSE244832, 61.5% in GSE136103) (Figure 7D,E). We conclude that the receptor-defined macrophage-to-HSC PDGF topology (HSC-enriched *PDGFRB*) was conserved across both external cohorts. The ligand-driven macrophage-to-HSC edge was replicated in the snRNA-seq cohort (GSE244832), whereas in CD45-sorted scRNA-seq (GSE136103) it could not be assessed—rather than being refuted—because *PDGFC* fell below the expression-proportion detection threshold on that platform (classified ‘not assessable’ in Table 2). In that cohort, PDGFRB was instead engaged by autocrine HSC-derived ligands (MFGE8 and PDGFA), indicating that the receptor arm remained active even where the macrophage-derived PDGFC ligand was not captured (Table S21). The receptor side of the axis is therefore replicated in both external cohorts, while the complete ligand-driven edge currently rests on a single external snRNA-seq cohort; its translational weight should be interpreted with that limitation in mind.

To provide tissue-level spatial support for this axis, we examined an independent MASLD Visium spatial transcriptomics atlas (35 sections, of which 33 were evaluable for the neighbor-enrichment test; 48,196 quality-controlled spots) and tested whether *PDGFC*^+^ SAMac-high and *PDGFRB*^+^ HSC-high spots co-localized more than expected (Figure 4A,C–F). Under a stratified permutation null to control for local HSC-score density, we found that HSC-high neighbors were focally enriched around SAMac-high spots (median 1.26-fold; 26 of 33 evaluable sections enriched; *p* = 1.7 × 10^−4^), after removal of approximately 15% inflation detected under a naïve uniform null (Figure 4D,E; Supplemental Figure S5). This enrichment was robust across eight null-model configurations (Figure 4F). The relative spatial affinity was constitutive, rather than escalating with clinical stage (JT z = −0.10, *p* = 0.92), consistent with a spatially organized macrophage–HSC niche present across disease stages. These spatial data are consistent with, and provide tissue-level spatial support for, the macrophage → HSC PDGF axis, and address the absence of spatial validation noted among the study limitations. As Visium spots (approximately 55 µm) contain multiple cells, this co-localization reflects niche-level spatial proximity, rather than direct cell–cell ligand–receptor signaling, and does not by itself establish a functional interaction; hence, the modest effect size (1.26-fold under the stratified null) is reported.

## 3. Discussion

Translational exploitation of MASH fibrosis single-cell and single-nucleus transcriptomics data faces critical challenges, largely due to potential reproducibility risks that complicate target prioritization [22,23,24]. Here, we applied a reproducibility-focused, per-donor statistical framework across three independent cohorts to successfully separate robust, platform-independent pathological features from technical and analytical artifacts. This approach facilitated a rigorous evaluation of the macrophage-to-HSC interactome and delivered a cross-cohort-validated molecular framework that clarifies the therapeutic boundaries within the MASH fibrotic microenvironment.

A major finding of this study is the high reproducibility of the 249-gene SAMac program, which demonstrated consistent, disease-stage-dependent expansion across both discovery and external validation cohorts. This stable transcriptomic signature reinforces the critical role of SAMacs in driving MASH-related fibrogenesis. We identified key molecules of the broader SAMac program that represent functional components, rather than passive markers. For example, this phenotype, which extends beyond the 249-gene intrinsic signature, includes the canonical markers TREM2—a molecule heavily implicated in promoting the efferocytosis of lipid-loaded apoptotic hepatocytes under lipotoxic stress [25,26]—and *MS4A7*, which is driven by damaged steatotic hepatocytes and promotes cellular activation [27,28]. The previous literature has highlighted that target discovery directed toward dynamic, unstable immune subpopulations often underlies the unsatisfactory efficacy of pan-inflammatory interventions in clinical trials [29,30,31]. Our data support an alternative approach: establishing a core pathogenic identity of SAMacs that remains cohort-invariant, despite variation in cell-capture methodologies. The identified 249-gene framework could serve as a robust baseline for patient stratification and companion diagnostics in future macrophage-targeted clinical trials.

Cell–cell communication analysis at the interactome level prioritized the macrophage-to-HSC intercellular axis, centered on PDGFC to PDGFRB. Importantly, network topology evaluation identified PDGFRB as a major, high-degree hub within the HSC fibrogenic interaction network, highlighting its potential as a candidate anti-fibrotic therapeutic node. The PDGF system is recognized as a potent mitogen for myofibroblasts [32,33]; however, our study refines this understanding by demonstrating that the PDGFC to PDGFRB interface represents a robust signaling conduit that survives technical variations across independent patient populations. This finding addresses an important knowledge gap in recent reports of liver spatial transcriptomics. While advanced spatial studies have successfully separated structural compartments, like the scar-core and scar-interface [34,35,36], the exact micro-zonal mapping of focal ligands remains largely uncompleted [37,38,39], and conventional non-spatial workflows are susceptible to dilution effects that underestimate focal fibrotic niche signals [39]. Our per-donor analysis recovered a stable PDGFC–PDGFRB axis that may be less susceptible to dilution bias than bulk or pooled single-cell averages, supporting a rationale for evaluating PDGFRB-directed agents. Because PDGFRB is a well-precedented target, approved PDGFR-family kinase inhibitors such as nintedanib (approved for idiopathic pulmonary fibrosis and systemic sclerosis-associated interstitial lung disease) and imatinib (approved for chronic myeloid leukemia and gastrointestinal stromal tumors)—neither currently approved for a liver indication, so their application here would represent repurposing—and receptor- or ligand-neutralizing biologics provide a pharmacological entry point (Table S14) [40,41]. In an exploratory drug-repurposing analysis, we screened LINCS L1000 connectivity signatures for compounds predicted to reverse the SAMac and HSC-activation programs, including consensus reversers across both signatures and the positioning of reference anti-fibrotic drugs (Table S18). We emphasize, however, that our support for the PDGFC-PDGFRB axis is computational and correlational (network topology and cross-donor receptor-pathway coupling) rather than functional: the axis is a reproducibility-filtered candidate node whose causal role in fibrogenesis and whose therapeutic value remain to be established in loss- and gain-of-function and in vivo studies. Nintedanib in particular is a multi-kinase inhibitor whose antifibrotic activity is not PDGFRB-selective, so target-specific attribution will require selective tools.

In sharp contrast to the stability of the macrophage program, we found that a previously published global HSC activation module failed to validate in external cohort data. This notable divergence warrants careful biological and methodological interpretation. While a specific sub-axis governing core ECM structural proteins and RUNX1/2 transcription factors remained robustly preserved, the broader, global features of HSC activation appeared highly cohort-specific. Pathologically, this structural dissociation may reflect profound spatial heterogeneities within the MASH microenvironment. Recent mechanistic and multi-omic studies have supported RUNX1 and RUNX2 as candidate master regulators in activated hepatic stellate cells, where they co-regulate pro-fibrogenic transcriptional programs and directly promote fibrotic effector genes [21]. Compartment-resolved SCENIC transcription-factor regulon activity and the mapped transcription-factor-to-fibrosis-target regulatory edges, with per-donor TF–target and TF–stage coherence, are provided in Tables S13 and S16. Nevertheless, while mainstream HSC research remains largely focused on the canonical TGFB–SMAD axis and its immediate epigenetic effectors [42,43,44], how the RUNX family maintains functional homeostasis or responds to mechanical cues within the chronic scar niche remains insufficiently explored [45,46,47]. Our findings imply that treating HSC activation as a uniform, global phenotypic shift oversimplifies MASH pathogenesis. Rather, anti-fibrotic strategies may achieve higher translational fidelity by focusing on the robustly conserved, RUNX-associated core structural program. We note, however, that RUNX1 and RUNX2 are transcription factors for which no approved direct-acting agent was returned in our druggability annotation (Table S14); near-term therapeutic exploitation is therefore more likely to proceed indirectly, through upstream inputs such as PDGFRB signaling or through downstream ECM effectors, than through direct RUNX inhibition.

This methodological distinction is further illustrated by our critical re-evaluation of the AP-1/JUNB regulatory axis. Although the AP-1/JUNB axis is widely implicated as a master driver of cellular activation in enzymatic scRNA-seq studies [48,49,50], accumulating evidence indicates that the enzymatic dissociation of cells during RNA extraction can induce immediate-early and stress-response programs, including AP-1 family members such as *FOS* and *JUN*, thereby confounding interpretation of activation-associated transcriptional states [48,51,52]. This observation aligns with recent methodological warnings establishing that warm enzymatic dissociation protocols reproducibly trigger the massive, artifactual upregulation of immediate early genes, including AP-1 components such as *FOS* and *JUN*, in freshly isolated cells [49,53]. By demonstrating the absence of this signature in frozen tissue cohorts analyzed using methods that bypass enzymatic digestion, our study provides cross-platform evidence that helps unravel true disease biology from ex vivo artifacts. Because immediate-early/AP-1 activity is substantially regulated post-transcriptionally (phosphorylation-dependent) and snRNA-seq preferentially captures nuclear RNA while under-representing cytoplasmic immediate-early transcripts, this transcript-level evidence does not exclude protein- or phosphorylation-level AP-1 activation; therefore, direct protein and phospho-level validation is warranted before exclusion of AP-1 as a therapeutic target.

From a systems perspective, our validated set of conserved hepatic programs aligns well with emerging models of the spleen–liver axis in metabolic inflammation [13,54]. Chronic splenic immune remodeling, including the expansion of splenic monocyte reservoirs and pro-inflammatory lymphoid populations, provides a systemic inflammatory flux that traffics to the liver via CCR2-dependent recruitment and shapes macrophage polarization [55,56]. In this context, the reproducible SAMac signature may represent a stereotypic polarization state that is consistent with the transcriptional imprint of systemically recruited myeloid cells within the fibrotic liver niche, rather than a purely liver-autonomous activation event [9,57]. Similarly, the conserved RUNX sub-axis in HSCs could reflect a core transcriptional module that renders stellate cells more responsive to local paracrine cues such as PDGF under conditions of sustained systemic inflammatory pressure [58,59]. Viewed this way, the hepatic programs isolated through per-donor verification correspond to the downstream hepatic “landing zone” of extrahepatic immunometabolic drivers, in keeping with the view that MASH fibrosis emerges from coordinated inter-organ inflammatory circuitry rather than liver-restricted pathways [60]. We emphasize that this interpretive framework is supported by congruence with the existing literature, not directly demonstrated by our hepatic-only datasets.

Despite the strengths of our multi-cohort verification approach, certain limitations must be acknowledged. First, after implementing rigorous quality control and minimum cell-count thresholds to eliminate pseudoreplication, the healthy control group in the discovery cohort was restricted to two donors (three before removal of the pre-specified healthy-baseline outlier HL2). Because two donors cannot support a well-powered healthy-versus-disease baseline contrast, we pre-specified the disease-only spectrum (MASLD to advanced MASH) as the primary progression analysis and used the healthy baseline only in explicitly labeled sensitivity checks (Table S23; donor accounting in Table S24). Our estimates of the healthy-to-disease transition and of absolute baseline magnitudes are therefore provisional and will require larger, highly standardized control cohorts; the progression signals that hold within the disease-only spectrum are the generalizable findings of this study. Second, our computational interactome analysis relied on transcript abundance, which can introduce dropouts or capture-efficiency bias for low-abundance secreted ligands, as seen with the low recovery of *PDGFC* in the CD45-sorted scRNA-seq dataset (GSE136103), where *PDGFC* fell below the detection threshold. Consequently, the ligand arm of the macrophage-to-HSC PDGF edge could be assessed only in the snRNA-seq validation cohort (GSE244832), where it replicated, and could not be evaluated in GSE136103; the HSC-enriched *PDGFRB* placement replicated in both external cohorts, but the complete ligand-driven edge currently rests on a single external cohort, which tempers its translational weight. Third, although our external validation datasets spanned cohorts that were independent geographically and in terms of the platforms used for their generation, all data represent cross-sectional clinical snapshots. Future research incorporating prospective longitudinal cohorts would be highly advantageous to definitively capture the temporal kinetics and resolution dynamics of the identified axes. Fourth, as snRNA-seq preferentially captures nuclear RNA and under-represents cytoplasmic immediate-early transcripts, our transcript-level evidence against an AP-1/JUNB activation axis should be interpreted as an absence of nuclear-transcript support, rather than a definitive refutation, particularly as immediate-early activity is largely regulated at the post-transcriptional level; moreover, the snRNA-seq validation cohort (GSE244832) spanned NORMAL/MASL/MASH but not cirrhotic/late-stage disease; hence, protein- and phosphorylation-level assessment across the full disease range is required. Fifth, the datasets analyzed here are liver-restricted and do not include matched splenic samples; consequently, any contribution of spleen-derived monocytes or lymphoid subsets to the observed SAMac expansion and HSC programs is inferred from the broader literature rather than directly quantified in the present cohorts. Finally, while our framework provided in silico evidence of molecular conservation, downstream in vivo functional rescue experiments and high-resolution spatial multi-omics analyses are ultimately required to validate the exact spatial coordinates, protein-level translation, and therapeutic safety boundaries of the proposed PDGF and RUNX sub-networks.

## 4. Materials and Methods

### 4.1. Ethics

Ethical review and approval were waived for this study because it is a secondary computational analysis of de-identified, publicly available datasets (GSE202379, GSE136103, GSE244832) for which ethical approval and informed consent were obtained in the original studies [9,21,61]; no new human or animal data were generated.

### 4.2. Cohort Acquisition and Quality Control

Human liver sc/snRNA-seq datasets were harvested from the Gene Expression Omnibus (GEO) database (National Center for Biotechnology Information, National Library of Medicine, National Institutes of Health, Bethesda, MD, USA; https://www.ncbi.nlm.nih.gov/geo/, accessed on 6 June 2026). The GSE202379 dataset served as the discovery cohort [61]; it spans the continuous clinical spectrum of metabolic dysfunction-associated steatotic liver disease (MASLD), including healthy controls, MASLD without MASH, early MASH, and advanced MASH. To ensure downstream translational inference robustness, independent external validation was implemented across two large-scale datasets representing distinct isolation techniques: a cell-sorted, droplet-based scRNA-seq dataset, using RNA extracted with enzymatic methods (GSE136103) [9], and a multiregional snRNA-seq cohort (GSE244832) [21]. GSE136103 profiled CD45+ and CD45− non-parenchymal fractions by droplet scRNA-seq; the CD45+ arm favors immune-cell recovery, while non-leukocyte compartments such as HSCs can be under-represented, and some ligands (e.g., PDGFC) sparsely detected [9]. Accordingly, this cohort was used mainly to validate SAMac expansion and HSC-enriched *PDGFRB* placement, not as a definitive test of ligand-driven PDGF edges. Standard quality control filtering was enforced across all individual matrices. Droplets or nuclei characterized by low gene-detection thresholds, excessive mitochondrial read fractions, or potential doublet profiles were strictly excluded. Note that after applying these quality control procedures and enforcing a minimum cell-count threshold of ≥20 captured cells or nuclei in a specific cell compartment, to mitigate analytical bias, the healthy control group within the discovery cohort (GSE202379) was restricted to two donors; a third nominally healthy donor (HL2) was a pre-specified healthy-baseline outlier and was removed before trend testing. Because a two-donor baseline cannot support a well-powered healthy-versus-disease contrast, the disease-only spectrum (MASLD, early MASH, advanced MASH) was pre-specified as the primary progression analysis, with healthy-inclusive analyses reported as sensitivity checks (Tables S23 and S24). To address the limited efficiency of standard two-group comparisons when baseline subgroup sizes are small, the Jonckheere–Terpstra (JT) test was used to assess ordered ordinal trends across the full MASH progression spectrum, leveraging the prespecified stage ordering in a nonparametric multisample framework [19,62].

### 4.3. Data Normalization, Integration, and Dimensionality Reduction

To correct for platform-specific technical variance and donor-level batch effects, individual digital expression matrices were subjected to standard normalization and variance stabilization. Highly variable genes were selected based on standardized variance metrics across each dataset. Batch effect correction and data integration were performed using Harmony (harmonypy v2.0.0, Broad Institute of MIT and Harvard, Cambridge, MA, USA; via SCANPY [63] v1.11.4, scanpy.external.pp.harmony_integrate) with the donor as the batch key (random_state = 42, max_iter_harmony = 20), applied to the top principal components of the scaled 2000 highly variable gene space. Importantly, this integration and the resulting embedding were used only for visualization and unsupervised Leiden clustering (resolution = 1.0); all quantitative statistical inference reported below was performed on donor-level summaries (per-donor fractions, mean signature scores, or pseudobulk aggregates; Section 4.5 and Section 4.6) or, for intercellular communication modeling, on pooled cells (Section 4.7), independently of the integration step, so that donor-level conclusions did not depend on cross-donor integration. Principal component analysis was applied to the integrated highly variable gene space for linear dimensionality reduction. The top statistically significant principal components were retained to construct a nearest-neighbor graph, which served as the mathematical foundation for subsequent non-linear visualization and unsupervised clustering.

### 4.4. Unsupervised Clustering and Low-Dimensional Visualization

Unsupervised cell clustering was conducted using the Leiden community detection algorithm [64] (leidenalg v0.10.2 with igraph v0.11.9, Leiden University, Leiden, The Netherlands; via SCANPY v1.11.4), operating on the constructed shared-nearest-neighbor graph. A fixed resolution of 1.0 was used to capture biologically distinct cell populations while avoiding over-clustering. To visualize the intrahepatic cellular landscape in a low-dimensional space, uniform manifold approximation and projection (umap-learn v0.5.9.post2, Tutte Institute for Mathematics and Computing, Ottawa, ON, Canada; via SCANPY v1.11.4) was applied to the structural embeddings. Major cell lineages (including hepatocytes, endothelial cells, cholangiocytes, mesenchymal cells, and diverse immune compartments) were broadly segregated and projected onto the uniform manifold approximation and projection space to evaluate overall cluster topologies and inter-donor mixing.

### 4.5. Per-Donor Pseudobulk Aggregation

To circumvent the pervasive issue of pseudoreplication bias, where single cells from the same patient are erroneously treated as statistically independent observations, a rigorous per-donor statistical aggregation framework was implemented. For each individual cohort, cell-level digital counts were aggregated within specific cell compartments (macrophages and hepatic stellate cells) on a per-donor basis, shifting the unit of statistical analysis from individual cells to individual donors. To ensure reliable pseudobulk aggregation, donors with fewer than 20 captured cells or nuclei in a specific cell compartment were excluded from subsequent per-donor statistical evaluation. This standard cell-count threshold was implemented to balance donor retention with statistical robustness. Sensitivity analyses spanning per-donor thresholds of 10 to 50 cells indicated that the core conserved findings were not artifacts of this specific cutoff: the donor-level macrophage–HSC receptor-pathway coupling remained stable across thresholds of ≥10 to ≥30 cells per donor, whereas the disease-stage expansion of the conserved macrophage signature was robust in the primary analysis (≥20-cell threshold, disease-only three-stage ordering) but attenuated or lost at the ≥30-cell threshold (where the MASLD stage retained only one donor) under binary disease-only encodings, and at the most permissive ≥10-cell setting, where low-count donors contribute noisier pseudobulk estimates (Table S23). Two complementary per-donor aggregation modes were used. (i) For abundance and activation trend tests, each qualifying donor contributed a single value per metric: the SAMac (or activated-HSC) fraction of its compartment and the mean per-cell signature score across that donor’s compartment cells; these per-donor values (Table S6) were entered into the Jonckheere–Terpstra trend test. (ii) For differential expression, per-donor pseudobulk profiles were built by summing raw counts across each donor’s compartment cells and modeled with edgeR. Donor inclusion followed a stepwise funnel: of the 47 profiled discovery donors, 2 retained no macrophage nuclei after cell-type and purity quality control (45 remaining) and a further 20 fell below the ≥20-nucleus-per-donor threshold, leaving 25 macrophage donors (24 after removal of the pre-specified healthy-baseline outlier HL2; 22 in the disease-only primary set); for the HSC compartment, all 47 donors retained nuclei after quality control and 16 fell below the ≥20-nucleus threshold, leaving 31 HSC donors (30 after HL2 removal; 28 disease-only). Marker-panel pseudobulk differential expression repeated with donor HL2 excluded is reported in Table S7. The number of donors contributing to every group and analysis, and the number excluded at each step, is tabulated in Table S24.

### 4.6. Differential Expression Analysis and Non-Parametric Trend Testing

Instead of performing conventional binary differential expression tests on pooled cells, donor-level aggregated pseudobulk matrices were applied to track transcriptional kinetics. Continuous phenotypic trajectories tracking disease severity across ordered stages were evaluated using the non-parametric JT trend test. Because the discovery healthy baseline comprised only two usable donors, the disease-only ordering (MASLD, early MASH, advanced MASH) was pre-specified as the primary trend, and the healthy-inclusive four-stage ordering (Healthy, MASLD, early MASH, advanced MASH) was retained as a sensitivity analysis; both orderings, together with cell-count-threshold and HL2 sensitivity, are reported in Table S23. For each ordered analysis, the JT test provided the trend statistic (z) and its *p*-value, whereas Spearman’s rho is reported separately for pairwise donor-level associations; both were computed strictly on donor-level distributions. Where multiple hypotheses were tested, *p*-values were adjusted using the Benjamini–Hochberg false discovery rate (FDR) procedure. Genes exhibiting significant, monotonic expression shifts synchronized with MASH progression were cross-referenced across discovery and validation cohorts to define invariant, cohort-independent gene programs. The robustness of these trend analyses was assessed in three ways (Supplemental Figures S3–S5): the donor-level *PDGFC* substate pattern was re-tested under a linear mixed model with a per-donor random intercept (naïve intraclass correlation coefficient = 0.054); stage-adjusted cross-donor coupling was re-computed under a finer five-level ordinal stage recoding (partial ρ = 0.49 under both the coarse four-level and finer five-level codings; the two codings were themselves near-perfectly concordant, Spearman ρ = 0.995); and spatial neighbor-enrichment was evaluated against a density-stratified permutation null, in addition to a uniform null.

### 4.7. Intercellular Communication Modeling

Intercellular communication networks were modeled using the LIANA framework (v1.7.3) [65], which integrates a consensus of multiple ligand–receptor inference methods. Ligand–receptor inference was computed once per cohort on the pooled, log1p-normalized single-cell or single-nucleus expression matrix (use_raw = False), using the LIANA rank_aggregate consensus of CellPhoneDB, NATMI, and Connectome (n_perms = 1000; fixed random seed 42); CellPhoneDB permutation *p*-values defined significant interactions (*p* < 0.05). The minimum expression-proportion threshold (expr_prop, the fraction of cells in a group required to express a gene for it to be tested) was set to 0.01 in the discovery cohort, where inference was performed at the finer macrophage and HSC substate level with correspondingly small per-substate cell numbers, and to 0.10 in the two external validation cohorts (GSE244832 and GSE136103), where inference was performed at the broader macrophage and HSC compartment level with larger per-group cell numbers. Because these two settings differ in both analysis level and expr_prop threshold, LIANA magnitude and specificity rank-aggregate scores are calibrated within each analysis and are not directly comparable in absolute value across the discovery and validation cohorts; only within-analysis significance and relative rank are interpreted. In the discovery cohort, this pooled inference was run on 4902 cells drawn from four macrophage and HSC substates across 43 disease-stage donors (the four healthy-baseline donors were excluded from cross-talk inference, so these totals are lower than the 46-donor, 5442-nucleus discovery compartment counts (i.e., the 47 profiled donors minus the pre-specified outlier HL2); full per-analysis donor accounting is given in Table S24) and returned 23,704 ligand–receptor interactions (11,698 between the macrophage and HSC compartments), all of which are reported in full in Table S12; the curated interaction shortlist carried forward for architectural mapping (Table S12) additionally required CellPhoneDB *p* < 0.05 and excluded ligand–receptor pairs whose ligand was one of a pre-specified set of 20 abundant hepatocyte-secreted or plasma proteins prone to ambient spillover in single-nucleus droplets (ALB, TTR, SERPINA1, AHSG, RBP4, ORM1, AMBP, APOH, HP, TF, FGA, FGB, FGG, FGL1, and the apolipoproteins APOA1, APOA2, APOB, APOC1, APOC3 and APOE); PDGFC was not among these excluded ligands, so the reported macrophage-to-HSC PDGF axis was unaffected by this list-based ambient filter. Because LIANA scores are computed on pooled cells and are not themselves per-donor, we added a separate per-donor pervasiveness check to confirm that prioritized edges were not driven by a few donors: for each shortlisted pair, a donor was counted as supporting the interaction when at least 10% of that donor’s source cells expressed the ligand and at least 10% of its target cells expressed the receptor (minimum five cells per side); and the fraction of donors meeting this criterion is reported in Table S12 (for example, PDGFC-PDGFRB from SAMac to activated HSC in 21 of 22 donors, 95.5%; PDGFC-PDGFRA in 22 of 22). Only interactions that were significant in the pooled inference, pervasive across donors, and reproduced across independent patient populations and platforms were carried forward for architectural mapping.

### 4.8. Network Topology Construction and Druggability Annotation

Downstream network topologies were constructed by treating individual ligands and receptors as distinct nodes, with directed edges representing inferred communication scores derived from the LIANA framework. High-degree communication hubs within the macrophage-to-HSC interactome were identified using network centrality metrics, focusing specifically on nodes maintaining structural stability across platforms. To explore the translational potential of the structurally conserved components within these topologies, core network nodes were pharmacologically annotated using the Drug–Gene Interaction Database (DGIdb) [66], cross-referencing targeted genes against current preclinical and clinical small-molecule or monoclonal antibody candidates. All analyses used Python 3.11 (Python Software Foundation, Wilmington, DE, USA) with SCANPY v1.11.4 (Helmholtz Zentrum München, Munich, Germany), anndata v0.12.1 (Helmholtz Zentrum München, Munich, Germany), LIANA v1.7.3 (Heidelberg University, Heidelberg, Germany), SciPy v1.15.0 (core pipeline) and v1.13.1 (spatial and robustness analyses) (SciPy Developers/NumFOCUS, Austin, TX, USA), and statsmodels v0.14.6 (statsmodels Developers/NumFOCUS, Austin, TX, USA). Differential expression used edgeR v4.4.0 (with limma v3.62.1; Walter and Eliza Hall Institute of Medical Research, Melbourne, VIC, Australia) in R v4.4.3 (R Foundation for Statistical Computing, Vienna, Austria). Protein–protein networks were built using STRING v12.0 (STRING Consortium, University of Copenhagen, Copenhagen, Denmark; https://string-db.org, accessed on 6 June 2026; interaction confidence ≥ 0.4). Druggability was annotated with the Drug–Gene Interaction Database (DGIdb) v5 (Washington University School of Medicine, St. Louis, MO, USA; https://dgidb.org, accessed on 6 June 2026). A fixed random seed (42) was used throughout for reproducibility.

## 5. Conclusions

In conclusion, our study highlights the value of adopting a “reproducibility-first” standard in single-cell translational genomics. By implementing a per-donor verification framework, we have reduced platform- and dissociation-dependent technical variation and assembled a reproducibility-filtered map of candidate MASH fibrogenesis pathways that was partially validated across independent cohorts. These reproducibility-filtered pathways offer more reliable starting points for hypothesis-driven, functionally validated therapeutic design, rather than validated drug targets in themselves, while our framework provides a constructive workflow for evaluating data robustness in future single-cell translational research.

## Figures and Tables

**Table 1:** Discovery and external validation cohorts.

Cohort	Role	Platform	Disease Axis	Macrophages	HSCs
GSE202379 *	Discovery	snRNA-seq	Healthy/MASLD/MASH/Advanced	2390 nuclei	3052 nuclei
GSE136103	External validation 1	scRNA-seq (CD45+/− sorted NPC)	Healthy vs. cirrhotic	10,851 cells	2396 cells
GSE244832	External validation 2	snRNA-seq	NORMAL/MASL/MASH	5743 nuclei	13,940 nuclei

Abbreviations: HSC, hepatic stellate cell; MASLD, metabolic dysfunction-associated steatotic liver disease; MASH, metabolic dysfunction-associated steatohepatitis; scRNA-seq, single-cell RNA sequencing; snRNA-seq, single-nucleus RNA sequencing. * The healthy arm of GSE202379 retained two donors after outlier removal following application of a ≥20-nuclei-per-donor threshold. Donor counts by group (GSE202379 discovery): 47 donors total (4 healthy, 7 MASLD, 27 MASH, 9 advanced); after the ≥20-nuclei-per-compartment threshold, per-donor analyses retained 25 macrophage donors (3 healthy, 3 MASLD, 13 MASH, 6 advanced) and 31 HSC donors (3 healthy, 5 MASLD, 16 MASH, 7 advanced), reduced to 2 usable healthy donors after removal of the pre-specified outlier HL2; the disease-only primary analysis used 22 macrophage and 28 HSC donors. The GSE136103 per-donor SAMac-fraction test used 5 healthy versus 5 cirrhotic donors. Full per-analysis donor accounting, including all sensitivity subsets, is provided in Table S24. Compartment nucleus counts in this table and Figure 1 (5442 total) exclude the pre-specified outlier HL2; the Table S5 substate totals include HL2.

**Table 2:** Three discovery findings tested across two independent external cohorts (validation verdict matrix).

Finding	Cohort (Role)	Test	Statistic	Verdict
F1: SAMac stage-expansion (Holds)	GSE136103 (scRNA-seq, PRIMARY)	SAMac fraction (within mac) cirrhotic vs. healthy	3.5% → 18.0%, MW *p* = 0.008	Replicated
F1: SAMac stage-expansion (Holds)	GSE244832 (snRNA-seq, secondary)	SAMac fraction by stage (JT)	n/a—snRNA-seq cannot resolve TREM2/CD9 surface markers	Not assessable (platform cannot resolve surface markers)
F1b: SAMac signature direction	GSE136103 (PRIMARY)	249-gene UP-fraction in ext SAMac (anchors excl.)	69.5% up, sign-test *p* = 6.4 × 10^−9^; rho = 0.29	Replicated
F8: SAMac 3-subset structure (Exploratory)	GSE136103 (PRIMARY)	Subset programs separate; NF-κB/TLR fraction by disease	subsets recovered; NF-κB 66% → 76% but MW *p* = 0.42	Partially supported (structure recovered; substate expansion n.s.)
F2/F3: HSC activation signature	GSE244832 (snRNA-seq, PRIMARY)	Score by stage (JT) + MASH-vs-NORMAL concordance	JT *p* = 0.77; non-circ concord rho = 0.02; UP-frac 50%	Not replicated (as a whole signature)
F2/F3: HSC activation signature	GSE136103 (scRNA-seq, secondary)	score cirrhotic vs. healthy + concordance	MW *p* = 0.99; UP-frac 48%	Not replicated (as a whole signature)
F2c: ECM/RUNX HSC sub-axis	GSE244832 (PRIMARY)	*RUNX1*/2, *EFEMP1*, *LTBP2* MASH vs. NORMAL (per-donor)	*EFEMP1* +2.1 FDR = 0.019; *RUNX2* +1.4 FDR = 0.019; *RUNX1* +0.9 nominal *p* = 0.009, FDR = 0.060; *LTBP2* +1.9 nominal *p* = 0.0145, FDR = 0.069	Replicated (consistent with the original report [21])
F9/F10: AP-1/***JUNB*** HSC driver	GSE244832 (PRIMARY)	*JUNB*/*JUN*/*FOS*/*KLF9* MASH vs. NORMAL (per-donor)	AP-1 flat-to-DOWN; *JUNB p* = 0.78, *KLF9 p* = 0.18 (no transcript-level support; possible dissociation-associated IEG signal, see Discussion)	Not replicated (no transcript-level support)
F5: PDGF axis topology (Exploratory)	Both cohorts	PDGFC localization in macrophages; PDGFRA/B localization in HSCs	*PDGFRB* 35.2% (GSE244832)/61.5% (GSE136103) HSC vs. ~0% mac; *PDGFC* mac-enriched in GSE244832 (below detection threshold in GSE136103)	Replicated (placement)
F5b: PDGFC → PDGFRB mac → HSC edge	GSE244832 (PRIMARY)	LIANA rank_aggregate	PDGFC → PDGFRB cellphone_ *p* < 0.001; RRA specificity 0.003, magnitude 0.40 (consensus scores interpretable as *p*-values, not percentiles); 175/748 by magnitude	Replicated
F5b: PDGFC → PDGFRB mac → HSC edge	GSE136103 (secondary)	LIANA rank_aggregate	*PDGFC* below expr threshold (sparse in CD45-sorted scRNA-seq); PDGFRB autocrine recovered	Not assessable (ligand below detection threshold)

Abbreviations: FDR, false discovery rate; IEG, immediate early gene; JT, Jonckheere–Terpstra; HSC, hepatic stellate cell; mac, macrophage; MASH, metabolic dysfunction-associated steatohepatitis; MW, Mann–Whitney; n/a, not applicable; n.s., not significant; SAMac, scar-associated macrophage; scRNA-seq, single-cell RNA sequencing; snRNA-seq, single-nucleus RNA sequencing. Verdict categories: Replicated, the finding held in the validation cohort; Partially supported, some but not all components of the finding held; Not replicated, the finding was testable but did not hold; Not assessable, the finding could not be tested on that platform (e.g., cell-surface markers unresolved by snRNA-seq, or ligand below the detection threshold).

## Data Availability

All datasets analyzed in this study are publicly available from the Gene Expression Omnibus: GSE202379 (discovery), GSE136103 and GSE244832 (external validation); the MASLD Visium spatial transcriptomics atlas is available from the National Genomics Data Center (NGDC/CNCB) OMIX repository under accession OMIX010136 (file OMIX010136-02; BioProject PRJCA025928; accessed on 28 August 2026). Analysis code and processed signatures are provided as Supplementary Materials.

## Supplementary Materials

The following supporting information can be downloaded at: https://www.mdpi.com/article/10.3390/ijms27177920/s1.

## Abbreviations

AP-1	Activator protein 1
CCR2	C-C motif chemokine receptor 2
DGIdb	Drug–Gene Interaction Database
ECM	Extracellular matrix
edgeR	Bioconductor package for differential expression analysis of count data
FDR	False discovery rate
HSC	Hepatic stellate cell
IEG	Immediate-early gene
JT	Jonckheere–Terpstra (test)
LIANA	Ligand–receptor ANalysis frAmework
MAPK/ERK	Mitogen-activated protein kinase/extracellular signal-regulated kinase
MASH	Metabolic dysfunction-associated steatohepatitis
MASLD	Metabolic dysfunction-associated steatotic liver disease
MW	Mann–Whitney (U test)
NPC	Non-parenchymal cell
PDGF	Platelet-derived growth factor
***PDGFC***	Platelet-derived growth factor C
***PDGFRA***	Platelet-derived growth factor receptor alpha
***PDGFRB***	Platelet-derived growth factor receptor beta
PI3K/AKT	Phosphoinositide 3-kinase/protein kinase B
***RUNX1***/2	RUNX family transcription factor 1 and 2
SAMac	Scar-associated macrophage
scRNA-seq	Single-cell RNA-sequencing
snRNA-seq	Single-nucleus RNA-sequencing
STRING	Search Tool for the Retrieval of Interacting Genes/proteins
TGFB–SMAD	Transforming growth factor beta/SMAD signaling
UMAP	Uniform manifold approximation and projection
Visium	10× Genomics Visium spatial transcriptomics platform
