# Supplementary material for: A Conserved Macrophage-to-Hepatic Stellate Cell PDGF Axis in Human MASH Identified by Multi-Cohort sc/snRNA-Seq Re-Analysis
Source: Int J Mol Sci. 2026 Sep 5;27(17):7920. doi: 10.3390/ijms27177920 (PMC13566530; doi:10.3390/ijms27177920)
Supplement: Supplementary file 1 [file ijms-27-07920-s001.zip › supplementary_figures/MASH_supplementary materials.pdf]

Supplementary Materials

Resolving replicable macrophage–HSC fibrotic crosstalk in human MASH: donor-level snRNA-seq reanalysis — supplementary exploratory and robustness analyses

These analyses are exploratory or robustness checks that support, but are not required for, the main-text conclusions. Main-text references to these figures are labelled accordingly.

Figure S1. SAMac substate trajectory and PDGFC expression (exploratory)

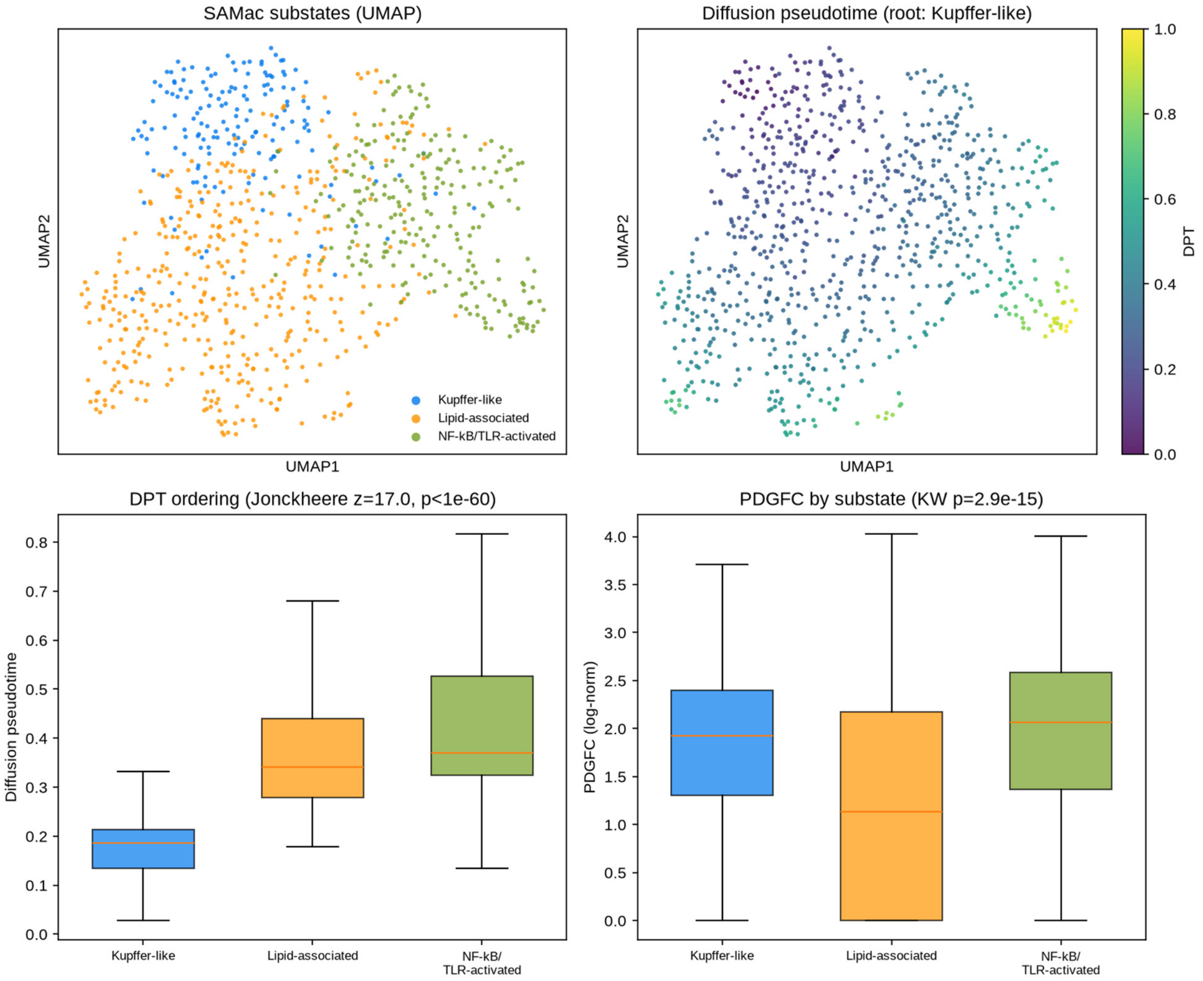

Figure S1. ***Scar-associated macrophage substate ordering and the PDGFC U-shape (exploratory).*** (A) UMAP of 823 SAMac nuclei (37 donors) coloured by the three transcriptional substates (Kupffer-like n=165, lipid-associated n=422, NF-κB/TLR-activated n=236). (B) Diffusion pseudotime (DPT) with a Kupffer-like root. (C) DPT increases monotonically across Kupffer-like < lipid-associated < NF-κB/TLR-activated (Jonckheere–Terpstra z=17.0, p=6.7×10^{-65}). (D) PDGFC (log-normalised) differs across substates (Kruskal–Wallis H=66.9, p=2.9×10^{-15}), with the lipid-associated

substate forming a relative trough (means: Kupffer-like 1.74, lipid-associated 1.12, NF-κB/TLR 1.83). This trajectory is exploratory and is not used to assert a directional differentiation sequence.

Figure S2. Functional submodule decomposition of the HSC activation module (exploratory)

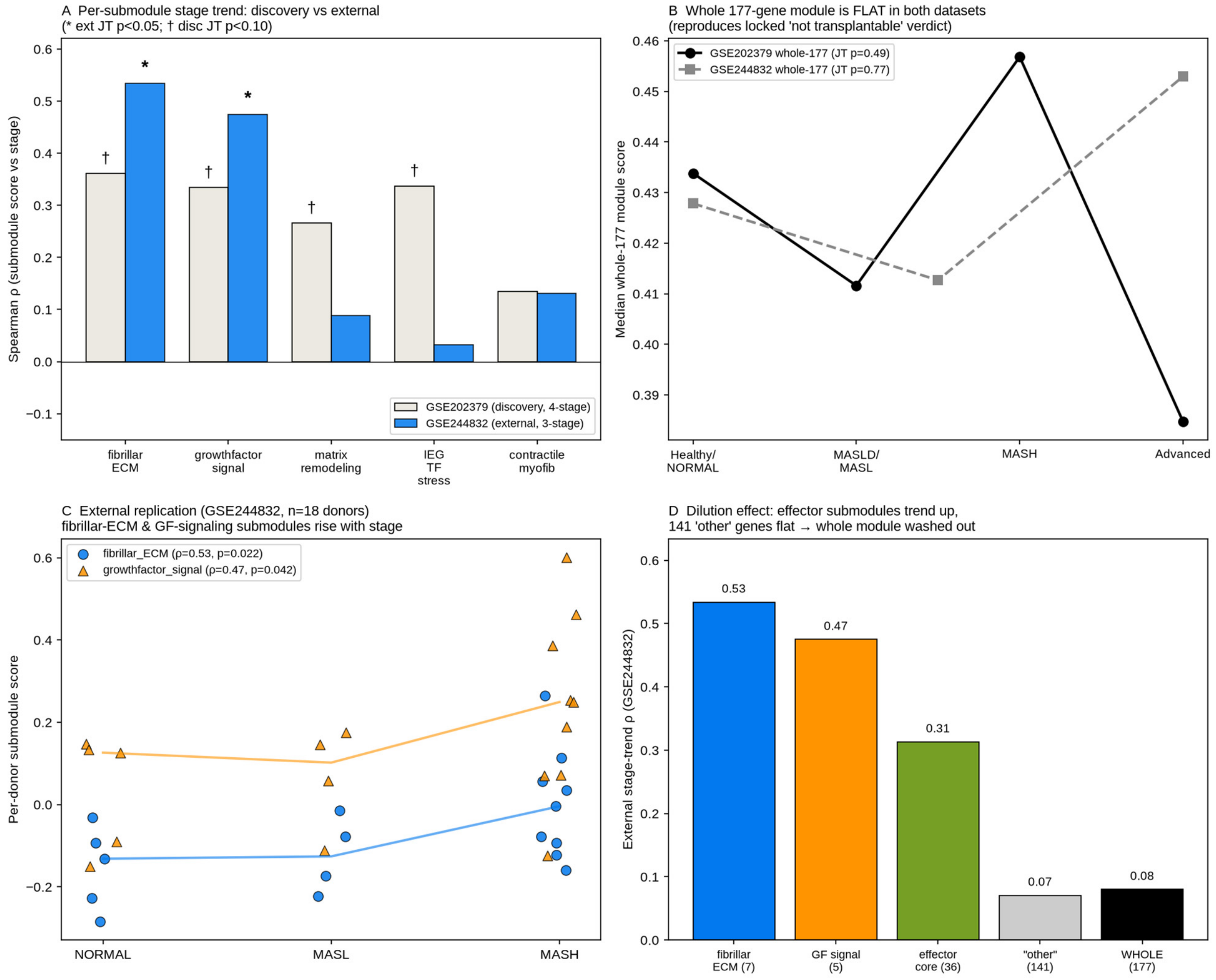

Figure S2. ***Submodule decomposition of the 177-gene HSC module (exploratory).*** (A) Per-submodule stage-trend Spearman ρ in discovery (GSE202379, 4 stages) versus external (GSE244832, 3 stages). (B) The whole 177-gene module is flat in both datasets (GSE202379 JT p=0.49; GSE244832 JT p=0.77), reproducing the locked “not transplantable” verdict. (C) In the external cohort (18 donors), the fibrillar-ECM (7 genes; ρ=0.53, nominal p=0.022) and growth-factor-signalling (5 genes; ρ=0.47, nominal p=0.042) submodules rise with stage. (D) A dilution model: effector submodules trend up while the 141 remaining genes are flat, washing out the whole-module signal. Important caveat: after Benjamini–Hochberg correction across submodules, no submodule survives FDR<0.05 (best FDR≈0.10); these submodule associations are therefore reported as nominal/exploratory only and do not modify the main-text per-gene edgeR result (EFEMP1, RUNX2; FDR=0.019).

Figure S3. Mixed-effects confirmation of the PDGFC substate pattern (robustness)

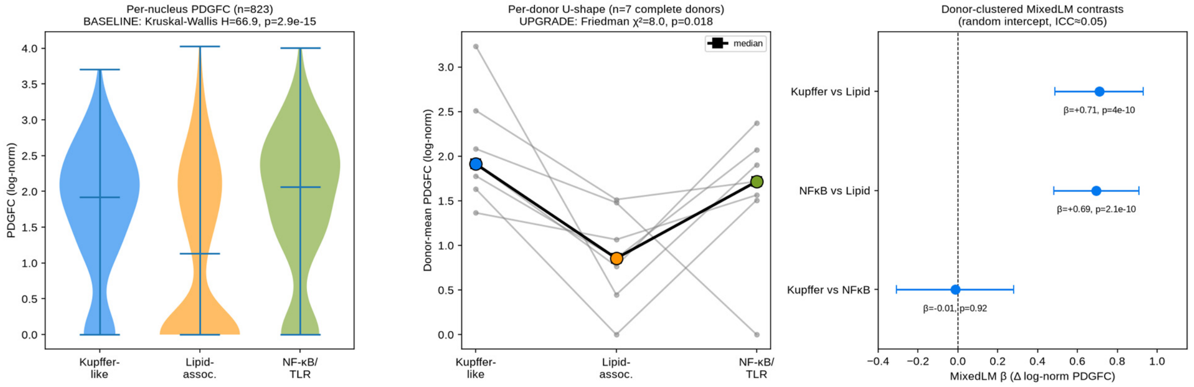

Figure S3. ***Donor random-intercept mixed model for PDGFC across SAMac substates (robustness).*** A linear mixed model (PDGFC ~ substate + (1|donor), REML) controls for donor pseudoreplication. The lipid-associated trough is preserved: Kupffer-like vs lipid-associated β=+0.71 (95% CI 0.49–0.93, $p=4.0\times10^{-10}$); NF-κB/TLR vs lipid-associated β=+0.70 ($p=2.1\times10^{-10}$); Kupffer-like vs NF-κB/TLR not different (p=0.92). Donor variance is modest (naive ICC=0.054), and a donor-level pseudobulk Friedman test is concordant. The U-shape is thus not an artefact of unequal per-donor nucleus contributions.

Figure S4. Stage-coding robustness of the cross-donor coupling (robustness)

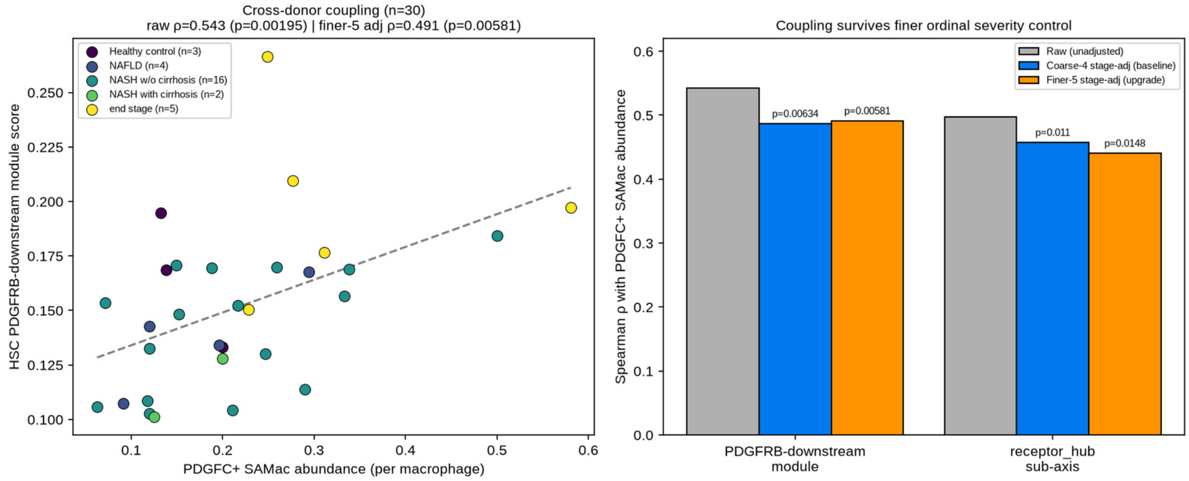

Figure S4. ***Finer 5-level ordinal stage recoding of the macrophage–HSC coupling (robustness).*** The coarse 4-level fibrosis staging was refined to a 5-level ordinal scheme (HC=3, NAFLD=4, NASH-without-cirrhosis=16, NASH-with-cirrhosis=2, end-stage=5; the two codings are monotonically concordant, ρ=0.995). The stage-adjusted partial correlation between $PDGFC^{+}$ SAMac load and the HSC PDGFRB-downstream score is unchanged (coarse-4 partial ρ=0.487 → finer-5 partial ρ=0.491, p=0.006; receptor-hub sub-axis 0.458 → 0.441, p=0.015). The coupling is therefore not an artefact of the specific stage discretisation.

Figure S5. Stratified-null spatial enrichment (robustness)

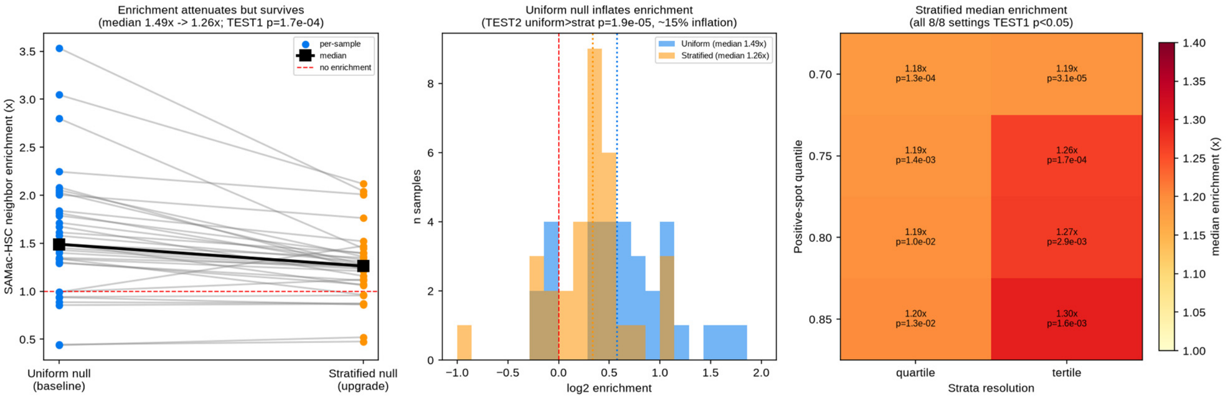

Figure S5. ***Stratified permutation null for the Visium spatial co-localization (robustness).*** Standalone version of the null-model comparison summarised in main-text Figure 7D–F. (Left) Per-section enrichment attenuates from a uniform null (median 1.49×) to a stratified null that shuffles HSC-high labels within HSC-score tertiles (median 1.26×; TEST1 $p=1.7\times10^{-4}$). (Middle) The uniform null overstates enrichment by ~15% (paired TEST2 Wilcoxon $p=1.9\times10^{-5}$). (Right) The stratified median enrichment stays significant across all 8/8 quantile×stratification settings (1.18–1.30×, all $p<0.05$). The residual ~1.26× is interpreted as genuine focal co-localization after removing the density-driven inflation.

Supplementary Tables

All supplementary tables are numbered consecutively S1–S24 without lettered sub-tables. Tables formerly split into lettered parts (e.g., Table S12b/S12c) are consolidated into their parent tables, with the original parts identifiable via the 'table_section' column or dedicated flag columns; the old-to-new mapping is recorded in SUPPLEMENTARY_TABLES_INDEX.csv of the source-data package. Tables with ≤60 rows are reproduced in full below; larger tables are provided as CSV files under supplementary_tables/ in the source-data package and as one sheet per table in source_data.xlsx.

Table S1. Bulk RNA-seq differential expression (limma), MASH vs. healthy, with post-ComBat batch-correction QC.

(Full table: 15246 rows — provided as supplementary_tables/TableS1_bulk_limma_DE.csv in the source-data package and as sheet S1 of source_data.xlsx.)

Table S2. MCP-counter immune/stromal population abundances (bulk deconvolution).

population	HC_median	MASH_median	log2FC_MASHvsHC	p_MannWhitney	padj_BH
Neutrophils	8.764	8.563	-0.0334	1.603e-07	1.603e-06
B lineage	6.672	6.802	0.02796	5.995e-05	0.0002311
T cells	6.95	7.136	0.03816	6.934e-05	0.0002311
Monocytic lineage	9.474	9.631	0.02384	0.001345	0.003362
Cytotoxic lymphocytes	7.314	7.483	0.0329	0.00331	0.006621
Fibroblasts	9.517	9.726	0.03136	0.01052	0.01754
Myeloid dendritic cells	7.217	7.315	0.0196	0.03091	0.04416
Endothelial cells	8.374	8.316	-0.009974	0.1241	0.1552
NK cells	7.042	7.012	-0.006045	0.8093	0.8992
CD8 T cells	7.288	7.433	0.0284	0.9505	0.9505

Table S3. T-cell pseudobulk differential expression (edgeR).

(Full table: 7250 rows — provided as supplementary_tables/TableS3_Tcell_pseudobulk_DE_edgeR.csv in the source-data package and as sheet S3 of source_data.xlsx.)

Table S4. Hub-gene differential expression in the augmented bulk cohort (edgeR + DESeq2) and augmented-cohort DEGs.

table_section	hub_gene	edgeR_log2FC	edgeR_pval	edgeR_FDR	DESeq2_log2FC	DESeq2_pval	DESeq2_padj	direction	verdict	gene	logFC	PValue	FDR
hub_gene_DE_edgeR_DESeq2	CD3D	0.341	0.3773	0.998	0.446	0.3046	1	up in MASH	direction-consistent but FDR-NS				
hub_gene_DE_edgeR_DESeq2	CD52	1.935	0.01827	0.998	1.998	0.01801	1	up in MASH	direction-consistent but FDR-NS				
hub_gene_DE_edgeR_DESeq2	CCL5	0.956	0.08115	0.998	0.883	0.1147	1	up in MASH	direction-consistent but FDR-NS				
hub_gene_DE_edgeR_DESeq2	IL7R	0.737	0.08268	0.998	0.83	0.03838	1	up in MASH	direction-consistent but				

									FDR-NS				
hub_gene_DE_edgeR_DESeq2	CD3G	0.646	0.07309	0.998	0.766	0.04711	1	up in MASH	direction-consistent but FDR-NS				
hub_gene_DE_edgeR_DESeq2	CD3E	0.656	0.04328	0.998	0.754	0.03015	1	up in MASH	direction-consistent but FDR-NS				
hub_gene_DE_edgeR_DESeq2	CD8A	0.771	0.1513	0.998	0.595	0.287	1	up in MASH	direction-consistent but FDR-NS				
augmented_cohort_DEGs_edgeR										LINC01524	-3.784	2.03e-08	0.0001317
augmented_cohort_DEGs_edgeR										MTRNR2L8	-3.608	2.531e-07	0.0008208
augmented_cohort_DEGs_edgeR										HSPA1B	-2.657	6.451e-06	0.01395
augmented_cohort_DEGs_edgeR										AC243829.1	-1.947	1.335e-05	0.01904
augmented_cohort_DEGs_edgeR										AC105402.3	-3.126	1.467e-05	0.01904

Table S5. Per-donor macrophage and HSC nuclei counts and donor feasibility summaries. The donor_feasibility_by_threshold block uses raw pre-QC per-donor counts, whereas donor_feasibility_by_threshold_per_stage uses the post-QC analysis pool (consistent with Table S24). Substate cell-count totals include the pre-specified outlier HL2.

(Full table: 202 rows — provided as supplementary_tables/TableS5_per_donor_counts_feasibility.csv in the source-data package and as sheet S5 of source_data.xlsx.)

Table S6. Stage-trend statistics for macrophage/HSC activation (Jonckheere-Terpstra + Kruskal-Wallis) with per-donor activation scores. Four-stage tests here retain donor HL2 (healthy n = 3); HL2-removed sensitivity values are given in Tables S8 and S10.

table_section	compartment	metric	JT_z_4stage	JT_p_4stage	KW_p_4stage	JT_z_diseaseonly	JT_p_diseaseonly	median_Healthy	median_MASLD	median_MASH	median_Advanced	verdict	Patient.ID	n_cells	mean_activation_score	activated_fraction	stage4	binary
stage_trend_statistics	Macrophage	mean_activation_score	2.481	0.0131	0.097	2.101	0.0356	-0.029	-0.037	0.005	0.053	Holds (significant monotonic increase)						
stage_trend_statistics	Macrophage	activated_fraction	2.736	0.0062	0.0637	2.23	0.0257	0.185	0.227	0.298	0.403	Holds (significant monotonic increase)						
stage_trend_statistics	HSC	mean_activation_score	0.984	0.3253	0.2531	1.808	0.0706	0.063	-0.015	-0.002	0.032	NS / Needs revision						
stage_trend_statistics	HSC	activated_fraction	0.724	0.4692	0.2508	1.607	0.1081	0.362	0.237	0.278	0.326	NS / Need						

												s revision						
per_donor_activation_scores	Macrophage												6	25	-0.08218	0.12	MASLD	MASLD
per_donor_activation_scores	Macrophage												8	71	0.03763	0.4225	MASH	MASH
per_donor_activation_scores	Macrophage												9	75	-0.02859	0.16	MASH	MASH
per_donor_activation_scores	Macrophage												16	25	-0.02066	0.28	MASH	MASH
per_donor_activation_scores	Macrophage												20	85	-0.04888	0.1647	MASH	MASH
per_donor_activation_scores	Macrophage												21	77	0.01096	0.3766	MASH	MASH
per_donor_activation_scores	Macrophage												49	25	-0.05258	0.2	MASH	MASH
per_donor_activation_scores	Macrophage												50	46	-0.02129	0.2609	MASH	MASH
per_donor_activation_scores	Macrophage												51	46	0.07207	0.3913	MASLD	MASLD
per_donor_activation_scores	Macrophage												56	22	-0.03672	0.2273	MASLD	MASLD
per_donor_activation_scores	Macrophage												57	47	0.004729	0.2979	MASH	MASH
per_donor_activation_scores	Macrophage												70	27	0.0306	0.4444	MASH	MASH
per_donor_activation_scores	Macrophage												72	203	0.02159	0.3251	MASH	MASH
per_donor_activation_scores	Macrophage												73	114	0.07638	0.4035	MASH	MASH
per_donor_activation_scores	Macrophage												75	35	0.05674	0.3714	Advanced	MASH
per_donor_activation_scores	Macrophage												76	117	-0.05593	0.2308	MASH	MASH
per_donor_activation_scores	Macrophage												77	27	0.0787	0.5185	MASH	MASH
per_donor_activation_scores	Macrophage												98	60	-0.005434	0.2833	Healthy	HC
per_donor_activation_scores	Macrophage												CL16	155	0.2349	0.7419	Advanced	MASH
per_donor_activation_scores	Macrophage												CL17	74	0.04912	0.4324	Advanced	MASH
per_donor_activation_scores	Macrophage												CL18	215	0.03535	0.3395	Advanced	MASH
per_donor_activation_scores	Macrophage												CL103	314	0.05968	0.4236	Advanced	MASH
per_donor_activation_scores	Macrophage												CL104	123	0.004428	0.3821	Advanced	MASH
per_donor_activation_scores	Macrophage												HL1	189	-0.04069	0.1852	Healthy	HC
per_donor_activation_scores	Macrophage												HL2	65	-0.02915	0.1846	Healthy	HC
per_donor_activation_scores	HSC												6	78	-0.04543	0.1923	MASLD	MASLD
per_donor_activation_scores	HSC												7	51	0.03132	0.2745	MASH	MASH
per_donor_activation_scores	HSC												8	95	0.1535	0.4842	MASH	MASH

per_donor_activation_scores	HSC												9	139	-0.05013	0.223	MASH	MASH
per_donor_activation_scores	HSC												15	20	-0.09413	0.15	MASH	MASH
per_donor_activation_scores	HSC												16	51	-0.001332	0.3137	MASH	MASH
per_donor_activation_scores	HSC												19	30	-0.06355	0.1667	MASH	MASH
per_donor_activation_scores	HSC												20	110	-0.01778	0.2818	MASH	MASH
per_donor_activation_scores	HSC												21	146	0.03914	0.3904	MASH	MASH
per_donor_activation_scores	HSC												50	80	-0.01272	0.2375	MASH	MASH
per_donor_activation_scores	HSC												51	76	-0.00393	0.2368	MASLD	MASLD
per_donor_activation_scores	HSC												54	34	0.0128	0.3824	MASH	MASH
per_donor_activation_scores	HSC												55	48	0.007804	0.2917	MASLD	MASLD
per_donor_activation_scores	HSC												56	117	-0.01475	0.3077	MASLD	MASLD
per_donor_activation_scores	HSC												57	43	0.1164	0.4884	MASH	MASH
per_donor_activation_scores	HSC												60	30	-0.06283	0.1667	MASLD	MASLD
per_donor_activation_scores	HSC												64	37	0.03603	0.3784	MASH	MASH
per_donor_activation_scores	HSC												68	48	-0.02291	0.25	Advanced	MASH
per_donor_activation_scores	HSC												70	25	0.0002409	0.32	MASH	MASH
per_donor_activation_scores	HSC												72	171	-0.01712	0.2515	MASH	MASH
per_donor_activation_scores	HSC												73	138	-0.02322	0.2319	MASH	MASH
per_donor_activation_scores	HSC												75	86	0.03171	0.3256	Advanced	MASH
per_donor_activation_scores	HSC												76	31	-0.002403	0.2581	MASH	MASH
per_donor_activation_scores	HSC												98	32	-0.03202	0.25	Healthy	HC
per_donor_activation_scores	HSC												CL16	95	-0.01188	0.2632	Advanced	MASH
per_donor_activation_scores	HSC												CL17	133	0.09217	0.3609	Advanced	MASH
per_donor_activation_scores	HSC												CL18	237	-0.009266	0.2658	Advanced	MASH
per_donor_activation_scores	HSC												CL103	315	0.1608	0.4476	Advanced	MASH
per_donor_activation_scores	HSC												CL104	134	0.06875	0.3433	Advanced	MASH
per_donor_activation_scores	HSC												HL1	246	0.3168	0.6829	Healthy	HC
per_donor_activation_scores	HSC												HL2	58	0.06333	0.3621	Healthy	HC

Table S7. Marker-panel differential expression with donor HL2 excluded.

table_section	compartment	gene	within_logFC	within_PValue	within_FDR	aug_logFC	aug_PValue	aug_FDR	design	n_DEG_FDR05_lfc025	n_DEG_FDR05_lfc05	top_gene	top_logFC	top_FDR
marker_panel_DE	HSC	PDGFRB	0.6468	0.2334	0.975	0.6025	0.4438	0.9995						
marker_panel_DE	HSC	DCN	-0.3514	0.3033	0.9839	-0.3795	0.528	0.9995						
marker_panel_DE	HSC	COL1A1	0.1812	0.6101	0.9936	0.1563	0.7793	0.9995						
marker_panel_DE	HSC	COL3A1	-0.06579	0.8186	0.9948	-0.1008	0.8319	0.9995						
marker_panel_DE	HSC	RBP1	0.2289	0.5995	0.9936	0.2012	0.7942	0.9995						
marker_panel_DE	HSC	LRAT	-0.659	0.3269	0.9855	-0.6949	0.3045	0.9995						
marker_panel_DE	HSC	ACTA2	-2.096	0.0001733	0.1649	-2.138	0.0003484	0.3343						
marker_panel_DE	HSC	TAGLN	-1.835	0.003695	0.6662	-1.861	0.002708	0.9995						
marker_panel_DE	HSC	TIMP1	1.425	0.2742	0.9807	1.379	0.3352	0.9995						
marker_panel_DE	HSC	PLAUR				1.283	0.3493	0.9995						
marker_panel_DE	macrophage	CD68	0.7719	0.1001	0.9131	0.7508	0.4659	1						
marker_panel_DE	macrophage	MARCO	-1.099	0.009449	0.6615	-1.16	0.09772	1						
marker_panel_DE	macrophage	CD163	-0.4903	0.2231	0.9235	-0.5487	0.2428	1						
marker_panel_DE	macrophage	LYZ	0.4405	0.6231	0.9831	0.3218	0.7867	1						
marker_panel_DE	macrophage	CSF1R	-0.2164	0.4837	0.9725	-0.2837	0.5414	1						
marker_panel_DE	macrophage	ITGAM	0.1154	0.7211	0.9922	0.05424	0.8998	1						
marker_panel_DE	macrophage	VSIG4	-0.237	0.5544	0.9765	-0.313	0.5449	1						
marker_panel_DE	macrophage	CD5L	-0.2993	0.4346	0.9613	-0.3637	0.6423	1						
marker_panel_DE	macrophage	TREM2												
marker_panel_DE	macrophage	SPP1	5.356	0.01479	0.7246	6.896	0.1598	1						
marker_panel_DE	macrophage	GPNMB	0.497	0.4303	0.9613	0.4311	0.6242	1						
marker_panel_DE	macrophage	CD9	0.964	0.1922	0.9217	0.9018	0.2822	1						
marker_panel_DE	macrophage	FABP5	2.603	0.005512	0.5736	2.626	0.01085	1						
marker_panel_DE	macrophage	OLR1				0.1199	0.9196	1						
DEG_count_summary	Macrophage								within_noHL2	0	0	FXYD6	-2.16	0.0581
DEG_count_summary	Macrophage								augmented_noHL2	0	0	AC020909.2	-3.06	0.789
DEG_count_summary	HSC								within_noHL2	3	3	HSPA1B	-4.3	0.000142
DEG_count_summary	HSC								augmented_noHL2	3	3	HBA2	-5.03	0.00987

Table S8. HSC activation and SAMac gene signatures (pseudobulk edgeR) with HSC stage-trend readouts. The HSC stage-trend readouts exclude the pre-specified outlier HL2 (healthy n = 2), matching Table S10.

(Full table: 429 rows — provided as supplementary_tables/TableS8_activation_signatures.csv in the source-data package and as sheet S8 of source_data.xlsx.)

Table S9. STRING PPI hub genes per compartment and single-cell signature vs. bulk gene-set overlap.

(Full table: 101 rows — provided as supplementary_tables/TableS9_STRING_hubs_bulk_overlap.csv in the source-data package and as sheet S9 of source_data.xlsx.)

Table S10. Full stage-trend descriptives per compartment. All tests here exclude the pre-specified outlier HL2 (healthy n = 2); HL2-retained four-stage values are given in Table S6.

compartment	metric	n_Healthy	n_MASLD	n_MASH	n_Advanced	median_Healthy	CI_Healthy	median_MASLD	CI_MASLD	median_MASH	CI_MASH	median_Advanced	CI_Advanced	JT_z_4stage	JT_p_4stage	KW_p_4stage	JT_z_diseaseonly	JT_p_diseaseonly	linear_slope	linear_r	linear_p	quadratic_F	quadratic_p	shape	verdict
Macrophage	SAMac_fraction (non-circular composition)	2	3	13	6	0.2343	[0.185,0.283]	0.2273	[0.120,0.391]	0.2979	[0.231,0.404]	0.4028	[0.355,0.587]	2.501	0.0124	0.1038	2.23	0.0257	0.0795	0.499	0.0131	1.349	0.2584	monotonic	Holds
Macrophage	SAMac_score (panel-derived)	2	3	13	6	-0.0325	[-0.047,-0.018]	-0.0525	[-0.087,0.056]	-0.0013	[-0.039,0.020]	0.0398	[0.006,0.135]	2.336	0.0195	0.1325	2.101	0.0356	0.0343	0.458	0.0245	1.661	0.2115	monotonic	Holds
HSC	activated_fraction (non-circular composition)	2	5	16	7	0.4665	[0.250,0.683]	0.2368	[0.167,0.308]	0.2782	[0.237,0.378]	0.3256	[0.263,0.361]	1.081	0.2795	0.3272	1.607	0.1081	-0.0096	-0.071	0.7107	3.086	0.0903	biphasic/curved	Needs revision
HSC	canonical_panel_score (non-circular)	2	5	16	7	0.1898	[0.069,0.310]	0.1461	[-0.040,0.165]	0.1513	[0.102,0.181]	0.0854	[0.034,0.104]	-1.671	0.0946	0.1121	-1.674	0.0942	-0.0279	-0.322	0.0828	0.587	0.4503	monotonic	Needs revision

Table S11. Ordinal machine-learning association for compartment features.

compartment	feature	feat_type	circularity	model	n	spearman_rho	kendall_tau	c_index	perm_p	perm_null_mean	confound_note	loo_null_center	interpretation
HSC	n_cells ONLY (confound baseline)	baseline	confound	linear	28	0.192		0.595	0.0473		per-donor cell number tracks stage; magnitude-feature floor		confound floor: cell number predicts stage by itself
HSC	Multivariate	multivariate	non-circular	ridge	28	0.6967	0.5769	0.8722	0.0013	-0.0513	random-91gene	-0.0513	ridge null≈-

	sig-minus-panel (91 genes), ridge a=10.0										ctrl rho=+0.43, 2% beat obs -> mostly confound floor		0.05; high rho partly artifact+cell-number confound (see random-gene ctrl); NOT clean biology
HSC	Multivariate sig-minus-panel (91 genes), ridge a=100.0	multivariate	non-circular	ridge	28	0.5903	0.4882	0.815	0.0013	-0.1296	random-91gene ctrl rho=+0.43, 2% beat obs -> mostly confound floor	-0.1296	ridge null≈-0.05; high rho partly artifact+cell-number confound (see random-gene ctrl); NOT clean biology
HSC	Intrinsic sig mean (minus panel)	single	non-circular	linear	28	0.464	0.3653	0.7357	0.003	-0.3585		-0.3585	raw LOO rho downward-biased (single-feat null≈-0.38); use perm_p
HSC	Intrinsic sig mean (full)	single	near-circular(flag)	linear	28	0.457	0.3653	0.7357	0.0025	-0.3627		-0.3627	raw LOO rho downward-biased (single-feat null≈-0.38); use perm_p
HSC	Canonical fibrosis score (score_genes)	single	non-circular	linear	28	0.1613	0.1127	0.5727	0.1014	-0.3464		-0.3464	raw LOO rho downward-biased (single-feat null≈-0.38); use perm_p
HSC(common)	HSC-only (common donors)	single	non-circular	linear	20	0.362	0.275	0.6752	0.0355	-0.3493		-0.3493	raw LOO rho downward-biased (single-feat null≈-0.38); use perm_p
Joint	n_cells ONLY (confound baseline)	baseline	confound	linear	20	0.294		0.658	0.0759		per-donor cell number tracks stage; magnitude-feature floor		confound floor: cell number predicts stage by itself
Joint	Joint macro+HSC canonical	joint	non-circular	linear	20	0.4709	0.3823	0.7436	0.02	-0.2288		-0.2288	2-feat; modest gain over single but within confound floor
Macrophage	n_cells ONLY (confound baseline)	baseline	confound	linear	22	0.362		0.689	0.0147		per-donor cell number tracks stage; magnitude-feature floor		confound floor: cell number predicts stage by itself
Macrophage	Multivariate sig-minus-panel (246 genes), ridge a=10.0	multivariate	non-circular	ridge	22	0.4986	0.4134	0.7704	0.0227	-0.0788	random-246gene ctrl rho=+0.44, 28% beat obs -> CONFOUNDED (not signature-specific)	-0.0788	ridge null≈-0.05; high rho partly artifact+cell-number confound (see random-gene ctrl); NOT

													clean biology
Macrophage	Multivariate sig-minus-panel (246 genes), ridge a=100.0	multivariate	non-circular	ridge	22	0.4883	0.4021	0.763	0.016	-0.1236	random-246gene ctrl rho=+0.44, 28% beat obs -> CONFOUNDED (not signature-specific)	-0.1236	ridge null≈-0.05; high rho partly artifact+cell-number confound (see random-gene ctrl); NOT clean biology
Macrophage	Canonical SAMac score (score_genes)	single	non-circular	linear	22	0.3495	0.2888	0.6889	0.025	-0.3942	partial-out n_cells r=+0.32 p=0.15 (NS after adj)	-0.3942	raw LOO rho downward-biased (single-feat null≈-0.38); use perm_p
Macrophage	Intrinsic sig mean (full)	single	near-circular(flag)	linear	22	0.1156	0.0849	0.5556	0.1254	-0.3806		-0.3806	raw LOO rho downward-biased (single-feat null≈-0.38); use perm_p
Macrophage	Intrinsic sig mean (minus panel)	single	non-circular	linear	22	0.0707	0.0623	0.5407	0.1529	-0.3805		-0.3805	raw LOO rho downward-biased (single-feat null≈-0.38); use perm_p
Macrophage(common)	Macro-only (common donors)	single	non-circular	linear	20	0.3921	0.3286	0.7094	0.0205	-0.4109		-0.4109	raw LOO rho downward-biased (single-feat null≈-0.38); use perm_p

Table S12. Full LIANA ligand-receptor interaction results (GSE202379 discovery cohort) with curated shortlist flag and per-donor robustness. The accompanying CSV contains only the 11,698 macrophage–HSC cross-compartment edges (of 23,704 significant interactions in total).

(Full table: 11698 rows — provided as supplementary_tables/TableS12_LIANA_LR.csv in the source-data package and as sheet S12 of source_data.xlsx.)

Table S13. SCENIC transcription-factor regulon activity by compartment.

(Full table: 355 rows — provided as supplementary_tables/TableS13_SCENIC_regulons.csv in the source-data package and as sheet S13 of source_data.xlsx.)

Table S14. DGIdb druggability annotation of PDGF-axis and interaction genes (full + approved/curated subset flag).

(Full table: 635 rows — provided as supplementary_tables/TableS14_DGIdb.csv in the source-data package and as sheet S14 of source_data.xlsx.)

Table S15. SAMac subcluster markers, per-donor composition, composition stage-trend, PDGFC source attribution, and marker dispersion.

(Full table: 195 rows — provided as supplementary_tables/TableS15_SAMac_subclusters.csv in the source-data package and as sheet S15 of source_data.xlsx.)

Table S16. Transcription-factor to fibrosis-target edges with per-donor TF-target and TF-stage coherence.

compartment	TF	target	TF_FDR	TF_FDR_sig	edge_type	note	n_donors	TFact_vs_target_rho	p_ab	TFact_vs_stage_rho	p_as	target_vs_stage_rho	p_ts
macro	IRF8	GPNMB	0.0009569	True	FDR-sig driver		31	0.568	0.001	0.396	0.027	0.264	0.151
macro	NR3C1	CD44	9.537e-05	True	FDR-sig driver		31	0.333	0.067	0.289	0.115	0.086	0.646
macro	JUN	GPNMB	0.0006954	True	FDR-sig driver		31	0.503	0.004	0.208	0.262	0.264	0.151
macro	ATF3	CD9	0.005384	True	FDR-sig driver		31	0.227	0.22	0.309	0.09	0.195	0.292
macro	ATF3	PLAUR	0.005384	True	FDR-sig driver		31	0.567	0.001	0.309	0.09	0.482	0.006
macro	ERF	TAGLN	0.02716	True	FDR-sig driver		31	0.182	0.328	-0.178	0.337	0.271	0.141
macro	STAT6	FABP5	0.006589	True	FDR-sig driver		31	0.008	0.967	-0.417	0.02	-0.084	0.654
macro	STAT2	TAGLN	0.0003338	True	FDR-sig driver		31	0.154	0.407	-0.318	0.081	0.271	0.141
hsc	JUNB	TIMP1	0.0002038	True	FDR-sig driver		36	0.161	0.349	0.556	0	0.412	0.013
hsc	JUNB	COL1A1	0.0002038	True	FDR-sig driver		36	0.054	0.755	0.556	0	-0.218	0.202
hsc	ATF1	ACTA2	0.005276	True	FDR-sig driver		36	0.555	0	0.03	0.864	0.178	0.3
hsc	FOS	THBS1	0.02669	True	FDR-sig driver		36	0.346	0.039	0.618	0	0.207	0.226
hsc	NR2F2	PDGFRB	0.08935	False	suggestive (NOT FDR-sig)	reported as suggestive only	36	0.45	0.006	-0.186	0.276	-0.108	0.531
hsc	ELK3	PDGFRB	0.6386	False	suggestive (NOT FDR-sig)	reported as suggestive only	36	0.019	0.912	0.571	0	-0.108	0.531

Table S17. HSC stage-stratified differential expression, senescence marker panel, pathway enrichment, and per-donor module scores.

(Full table: 11779 rows — provided as supplementary_tables/TableS17_HSC_stage_stratified.csv in the source-data package and as sheet S17 of source_data.xlsx.)

Table S18. LINCS/CMap signature-reversal screen (SAMac and HSC signatures) with consensus reversers and reference-drug positioning.

(Full table: 1006 rows — provided as supplementary_tables/TableS18_LINCS_reversers.csv in the source-data package and as sheet S18 of source_data.xlsx.)

Table S19. HSC-arm external validation (per cohort/test).

Cohort	Compartment	Test	Method	Donor n	Statistic	Note	Verdict
GSE244832 (snRNA)	HSC (clusters 3+11)	Signature score by stage (per-donor mean)	Jonckheere–Terpstra	NORMAL/MASL/MASH	z=0.29, p=0.7712;	medians	NOT replicated

			trend	= 5/4/9 donors	KW p=0.6901	0.428/0.413/0.453	
GSE244832 (snRNA)	HSC	177-sig direction concordance, MASH vs NORMAL (non-circular)	Spearman rho + sign-test on UP-fraction	9 vs 5 donors (pseudobulk)	genome-wide rho=0.020 (p=0.097); 177-sig UP-frac 49.7% (p=0.562)	49.7% up = random	NOT replicated
GSE244832 (snRNA)	HSC	Sign concordance on 29 strong internal DEGs (non-circular)	Binomial sign-test	9 vs 5 donors	55.2% concordant (n=29, p=0.356)	—	NOT replicated
GSE244832 (snRNA)	HSC	Canonical contractile markers, MASH vs NORMAL	per-donor pseudobulk logFC	9 vs 5 donors	ACTA2 -0.12, TAGLN -0.52, PLAUR -0.19; TIMP1 +1.16, COL1A1 +0.33	contractile core flat-to-down	NOT replicated
GSE244832 (snRNA)	HSC	ECM/RUNX sub-axis: EFEMP1	Mann–Whitney (MASH>NORMAL) + BH-FDR over 19 genes	9 vs 5 donors	log2FC +2.11, p=0.00200, FDR=0.019	—	REPLICATED
GSE244832 (snRNA)	HSC	ECM/RUNX sub-axis: RUNX2	Mann–Whitney + BH-FDR	9 vs 5 donors	log2FC +1.38, p=0.00200, FDR=0.019	—	REPLICATED
GSE244832 (snRNA)	HSC	ECM/RUNX sub-axis: RUNX1	Mann–Whitney + BH-FDR	9 vs 5 donors	log2FC +0.93, p=0.0095, FDR=0.060	nominal	Partially replicated
GSE244832 (snRNA)	HSC	ECM/RUNX sub-axis: LTBP2	Mann–Whitney + BH-FDR	9 vs 5 donors	log2FC +1.92, p=0.0145, FDR=0.069	nominal	Partially replicated
GSE244832 (snRNA)	HSC	AP-1 family (JUNB/JUN/FOS/FOSB/JUND/ATF3/FOSL2)	Mann–Whitney + BH-FDR	9 vs 5 donors	all flat-to-DOWN; JUNB -0.34 (p=0.78), FOS -1.35, FOSB -3.56; all FDR>0.4	no transcript-level support; possible dissociation-associated IEG signal (see Discussion)	NOT replicated
GSE244832 (snRNA)	HSC	KLF9 / SERPINE1	Mann–Whitney + BH-FDR	9 vs 5 donors	KLF9 +0.52 (p=0.18, n.s.); SERPINE1 +0.004 (p=0.30, n.s.)	—	NOT replicated
GSE136103 (scRNA)	Mesenchyme (leiden 16+22)	177-sig score, cirrhotic vs healthy (secondary)	Mann–Whitney (cirr>healthy)	5 vs 5 donors	healthy med 0.279 > cirr 0.236; p=0.992; UP-frac 48% (p=0.716)	confirms null in 2nd cohort	NOT replicated

Table S20. SAMac / macrophage-arm external validation (per cohort/test).

Cohort	Compartment	Test	Method	Donor n	Statistic	Note	Verdict
GSE136103 (scRNA, PRIMARY)	Macrophage (leiden 0-3)	SAMac fraction within macrophages, cirrhotic vs healthy	Mann–Whitney (cirr>healthy); anchor TREM2+ & (CD9+|GPNMB+)	5 vs 5 donors	healthy 3.5% → cirrhotic 18.0%; U=24, p=0.0079	~5-fold expansion	REPLICATED
GSE136103 (scRNA, PRIMARY)	Macrophage	SAMac 249-gene signature direction (SAMac vs other mac), anchors excluded	Spearman rho + sign-test on UP-fraction	per-cluster pseudobulk	rho=0.286 (p=2.3e-05); UP-frac 69.5%, sign-test p=6.4e-09 (n=213)	FABP5/LGALS3/CTSD/CD9 concordant	REPLICATED
GSE136103 (scRNA, PRIMARY)	Macrophage	Mean SAMac score per donor (sort-confounded metric)	Mann–Whitney	5 vs 5 donors	healthy med 0.124 ≈ cirr 0.123; p=0.790	CD45-sort confounds abundance; use fraction metric	Not informative (confounded)
GSE136103 (scRNA, PRIMARY)	Macrophage	Kupffer (MARCO+|CD5L+) fraction, replacement check	Mann–Whitney	5 vs 5 donors	healthy 33.0% → cirrhotic 24.7%; p=0.111	directional KC→SAMac, n.s.	Directionally consistent (n.s.)
GSE136103 (scRNA, PRIMARY)	Macrophage	3-subset structure (Kupffer-like/Lipid/NF-κB-TLR)	Composition + NF-κB/TLR fraction MW	5 vs 5 donors	NF-κB/TLR 66.4%→76.4% (p=0.421); Kupffer-like 25%→13%; Lipid 8.6%→10.2%	programs separate; expansion n.s. at 5v5	Partially replicated (exploratory)

GSE244832 (snRNA, secondary)	Myeloid (cluster 5)	SAMac fraction by stage	Jonckheere–Terpstra	NORMAL/MASL/MASH 5/4/9 donors	all-stage median fraction 0.0; z=0.75, p=0.455	snRNA poorly captures TREM2/CD9/GPNMB surface markers	Not assessable (platform)

Table S21. Cross-cohort PDGF ligand/receptor placement and external LIANA recovery.

table_section	Cohort	Layer	Gene	Macrophage	HSC	Interpretation	Source→Target	Ligand→Receptor	CellPhoneDB p	Magnitude rank	Specificity rank	Rank by magnitude	Total interactions	Recovery
PDGF_ligand_receptor_placement	GSE244832 (snRNA)	Placement	PDGFC	Mac 39.5% (mean 0.899)	HSC 7.7% (mean 0.149)	ligand enriched in Mac								
PDGF_ligand_receptor_placement	GSE244832 (snRNA)	Placement	PDGFA	Mac 0.4% (mean 0.007)	HSC 5.6% (mean 0.107)	ligand below 10% detection threshold (not assessable)								
PDGF_ligand_receptor_placement	GSE244832 (snRNA)	Placement	PDGFRA	Mac 0.6% (mean 0.012)	HSC 14.8% (mean 0.290)	receptor enriched in HSC								
PDGF_ligand_receptor_placement	GSE244832 (snRNA)	Placement	PDGFRB	Mac 0.8% (mean 0.017)	HSC 35.2% (mean 0.810)	receptor enriched in HSC								
PDGF_ligand_receptor_placement	GSE136103 (scRNA)	Placement	PDGFC	Mac 8.9% (mean 0.091)	HSC 6.5% (mean 0.070)	ligand below 10% detection threshold (not assessable)								
PDGF_ligand_receptor_placement	GSE136103 (scRNA)	Placement	PDGFA	Mac 0.8% (mean 0.008)	HSC 35.5% (mean 0.602)	ligand in HSC								
PDGF_ligand_receptor_placement	GSE136103 (scRNA)	Placement	PDGFRA	Mac 0.2% (mean 0.002)	HSC 11.1% (mean 0.133)	receptor enriched in HSC								
PDGF_ligand_receptor_placement	GSE136103 (scRNA)	Placement	PDGFRB	Mac 0.4% (mean 0.004)	HSC 61.5% (mean	receptor enriched in HSC								

					1.059)									
PDGF_LIANA_recovery_external	GSE244832 (snRNA)						Macrophage→ HSC	PDGFC→PDGFRB	0	0.403	0.003	175	748	RECOVERED
PDGF_LIANA_recovery_external	GSE244832 (snRNA)						Macrophage→ HSC	PDGFC→PDGFRA	0	0.452	0.028	218	748	RECOVERED
PDGF_LIANA_recovery_external	GSE136103 (scRNA)						Macrophage→ HSC	PDGFC→PDGFRA/B				not assessable	951	Not assessable (ligand below detection threshold): PDGFC below 10% expr-prop in CD45-sorted scRNA; PDGFRB instead engaged by autocrine HSC ligands MFGE8/PDGFA

Table S22. Cross-cohort validation verdict matrix.

Finding	Cohort (role)	Test	Statistic	Verdict
F1: SAMac stage-expansion (Holds)	GSE136103 (scRNA, PRIMARY)	SAMac fraction (within mac) cirrhotic vs healthy	3.5%→18.0%, MW p=0.008	Replicated
F1: SAMac stage-expansion (Holds)	GSE244832 (snRNA, secondary)	SAMac fraction by stage (JT)	n/a — snRNA can't resolve TREM2/CD9 surface markers	Not assessable (platform cannot resolve surface markers)
F1b: SAMac signature direction	GSE136103 (PRIMARY)	249-gene UP-fraction in ext SAMac (anchors excl.)	69.5% up, sign-test p=6.4e-09; rho=0.29	Replicated
F8: SAMac 3-subset structure (Explor.)	GSE136103 (PRIMARY)	subset programs separate; NF-kB/TLR fraction by disease	subsets recovered; NF-kB 66%→76% but MW p=0.42	Partially supported (structure recovered; substate expansion n.s.)
F2/F3: HSC activation signature	GSE244832 (snRNA, PRIMARY)	score by stage (JT) + MASH-vs-NORMAL concordance	JT p=0.77; non-circ concord rho=0.02; UP-frac 50%	Not replicated (as a whole signature)
F2/F3: HSC activation signature	GSE136103 (scRNA, secondary)	score cirrhotic vs healthy + concordance	MW p=0.99; UP-frac 48%	Not replicated (as a whole signature)
F2c: ECM/RUNX HSC sub-axis	GSE244832 (PRIMARY)	RUNX1/2, EFEMP1, LTBP2 MASH vs NORMAL (per-donor)	EFEMP1 +2.1 FDR = 0.019; RUNX2 +1.4 FDR = 0.019; RUNX1 +0.9 nominal p = 0.009, FDR = 0.060; LTBP2 +1.9 nominal p = 0.0145, FDR = 0.069	Replicated (consistent with the original report [21])
F9/F10: AP-1/JUNB HSC driver	GSE244832 (PRIMARY)	JUNB/JUN/FOS/KLF9 MASH vs NORMAL (per-donor)	AP-1 flat-to-DOWN; JUNB p=0.78, KLF9 p=0.18 (no transcript-level support; possible dissociation-associated IEG signal, see Discussion)	Not replicated (no transcript-level support)
F5: PDGF axis topology (Explor.)	Both cohorts	PDGFC in mac? PDGFRA/B in HSC?	PDGFRB 35.2% (GSE244832) / 61.5% (GSE136103) HSC vs. ~0% mac; PDGFC mac-enriched in GSE244832 (below detection threshold in GSE136103)	Replicated (placement)
F5b: PDGFC→PDGFRB mac→HSC edge	GSE244832 (PRIMARY)	LIANA rank_aggregate	PDGFC→PDGFRB cellphone_p < 0.001; RRA specificity 0.003, magnitude 0.40 (consensus scores interpretable as p-values, not percentiles); 175/748 by magnitude	Replicated
F5b: PDGFC→PDGFRB mac→HSC edge	GSE136103 (secondary)	LIANA rank_aggregate	PDGFC below expr threshold (sparse in CD45-sorted scRNA); PDGFRB autocrine recovered	Not assessable (ligand below detection threshold)

Table S23. Sensitivity and robustness grid for the stage-trend analyses (Jonckheere-Terpstra).

trend	compartment	HL2_status	cell_threshold	n_donors	stage_coding	stage_inclusion	stages	n_per_stage	medians	direction	JT_z	JT_p	note	verdict
A_SAMac_fraction	Macrophage	with_HL2	20	25	4stage	all	Healthy/MASLD/MASH/Advanced	3/3/13/6	0.185/0.227/0.298/0.403	increase	2.736	0.0062	LOCKED HEADLINE z=2.74	holds (sig increase)
A_SAMac_fraction	Macrophage	with_HL2	20	25	4stage	disease_only	MASLD/MASH/Advanced	3/13/6	0.227/0.298/0.403	increase	2.23	0.0257		holds (sig increase)
A_SAMac_fraction	Macrophage	with_HL2	20	25	binary	all	HC/MASLD/MASH	3/3/19	0.185/0.227/0.371	increase	2.046	0.0407		holds (sig increase)
A_SAMac_fraction	Macrophage	with_HL2	20	25	binary	disease_only	MASLD/MASH	3/19	0.227/0.371	increase	1.292	0.1965		loses (NS)
A_SAMac_fraction	Macrophage	with_HL2	30	19	4stage	all	Healthy/MASLD/MASH/Advanced	3/1/9/6	0.185/0.391/0.298/0.403	increase	2.469	0.0135		holds (sig increase)
A_SAMac_fraction	Macrophage	with_HL2	30	19	4stage	disease_only	MASLD/MASH/Advanced	1/9/6	0.391/0.298/0.403	increase	1.709	0.0875		attenuates (trend, NS)
A_SAMac_fraction	Macrophage	with_HL2	30	19	binary	all	HC/MASLD/MASH	3/1/15	0.185/0.391/0.371	increase	1.342	0.1797		loses (NS)
A_SAMac_fraction	Macrophage	with_HL2	30	19	binary	disease_only	MASLD/MASH	1/15	0.391/0.371	decrease	-0.542	0.5876		loses (NS)
A_SAMac_fraction	Macrophage	with_HL2	50	15	4stage	all	Healthy/MASH/Advanced	3/7/5	0.185/0.325/0.424	increase	2.535	0.0112		holds (sig increase)
A_SAMac_fraction	Macrophage	with_HL2	50	15	4stage	disease_only	MASH/Advanced	7/5	0.325/0.424	increase	2.03	0.0424		holds (sig increase)
A_SAMac_fraction	Macrophage	with_HL2	50	15	binary	all	HC/MASH	3/12	0.185/0.379	increase	1.588	0.1124		attenuates (trend, NS)
A_SAMac_fraction	Macrophage	with_HL2	50	15	binary	disease_only	MASH	12	0.379	flat	0	1		loses (non-monotonic/NS)
A_SAMac_fraction	Macrophage	noHL2	10	33	4stage	all	Healthy/MASLD/MASH/Advanced	2/4/19/8	0.234/0.261/0.280/0.377	increase	1.468	0.1421		attenuates (trend, NS)
A_SAMac_fraction	Macrophage	noHL2	10	33	4stage	disease_only	MASLD/MASH/Advanced	4/19/8	0.261/0.280/0.377	increase	1.223	0.2214		loses (NS)
A_SAMac_fraction	Macrophage	noHL2	10	33	binary	all	HC/MASLD/MASH	2/4/27	0.234/0.261/0.325	increase	1.115	0.265		loses (NS)
A_SAMac_fraction	Macrophage	noHL2	10	33	binary	disease_only	MASLD/MASH	4/27	0.261/0.325	increase	0.766	0.4437		loses (NS)
A_SAMac_fraction	Macrophage	noHL2	20	24	4stage	all	Healthy/MASLD/MASH/Advanced	2/3/13/6	0.234/0.227/0.298/0.403	increase	2.501	0.0124		holds (sig increase)
A_SAMac_fraction	Macrophage	noHL2	20	24	4stage	disease_only	MASLD/MASH/Advanced	3/13/6	0.227/0.298/0.403	increase	2.23	0.0257		holds (sig increase)
A_SAMac_fraction	Macrophage	noHL2	20	24	binary	all	HC/MASLD/MASH	2/3/19	0.234/0.227/0.371	increase	1.728	0.0839		attenuates (trend, NS)
A_SAMac_fraction	Macrophage	noHL2	20	24	binary	disease_only	MASLD/MASH	3/19	0.227/0.371	increase	1.292	0.1965		loses (NS)
A_SAMac_fraction	Macrophage	noHL2	30	18	4stage	all	Healthy/MASLD/MASH/Advanced	2/1/9/6	0.234/0.391/0.298/0.403	increase	2.209	0.0272		holds (sig increase)
A_SAMac_fraction	Macrophage	noHL2	30	18	4stage	disease_only	MASLD/MASH/Advanced	1/9/6	0.391/0.298/0.403	increase	1.709	0.0875		attenuates (trend, NS)
A_SAMac_fraction	Macrophage	noHL2	30	18	binary	all	HC/MASLD/MASH	2/1/15	0.234/0.391/0.371	increase	0.884	0.3765		loses (NS)
A_SAMac_fraction	Macrophage	noHL2	30	18	binary	disease_only	MASLD/MASH	1/15	0.391/0.371	decrease	-0.542	0.5876		loses (NS)

A_SAMac_fraction	Macrophage	noHL2	50	14	4stage	all	Healthy/MASH/Advanced	2/7/5	0.234/0.325/0.424	increase	2.366	0.018		holds (sig increase)
A_SAMac_fraction	Macrophage	noHL2	50	14	4stage	disease_only	MASH/Advanced	7/5	0.325/0.424	increase	2.03	0.0424		holds (sig increase)
A_SAMac_fraction	Macrophage	noHL2	50	14	binary	all	HC/MASH	2/12	0.234/0.379	increase	1.278	0.2012		loses (NS)
A_SAMac_fraction	Macrophage	noHL2	50	14	binary	disease_only	MASH	12	0.379	flat	0	1		loses (non-monotonic/NS)
B_SAMac_score	Macrophage	with_HL2	20	25	4stage	all	Healthy/MASLD/MASH/Advanced	3/3/13/6	-0.029/-0.037/0.005/0.053	increase	2.481	0.0131	LOCKED z=2.48	holds (sig increase)
B_SAMac_score	Macrophage	with_HL2	20	25	4stage	disease_only	MASLD/MASH/Advanced	3/13/6	-0.037/0.005/0.053	increase	2.101	0.0356		holds (sig increase)
B_SAMac_score	Macrophage	with_HL2	20	25	binary	all	HC/MASLD/MASH	3/3/19	-0.029/-0.037/0.022	increase	1.606	0.1084		attenuates (trend, NS)
B_SAMac_score	Macrophage	with_HL2	20	25	binary	disease_only	MASLD/MASH	3/19	-0.037/0.022	increase	0.909	0.3634		loses (NS)
B_SAMac_score	Macrophage	with_HL2	30	19	4stage	all	Healthy/MASLD/MASH/Advanced	3/1/9/6	-0.029/0.072/0.005/0.053	increase	2.089	0.0367		holds (sig increase)
B_SAMac_score	Macrophage	with_HL2	30	19	4stage	disease_only	MASLD/MASH/Advanced	1/9/6	0.072/0.005/0.053	decrease	1.191	0.2337		loses (NS)
B_SAMac_score	Macrophage	with_HL2	30	19	binary	all	HC/MASLD/MASH	3/1/15	-0.029/0.072/0.022	increase	1.043	0.2967		loses (NS)
B_SAMac_score	Macrophage	with_HL2	30	19	binary	disease_only	MASLD/MASH	1/15	0.072/0.022	decrease	-1.193	0.2328		loses (NS)
B_SAMac_score	Macrophage	with_HL2	50	15	4stage	all	Healthy/MASH/Advanced	3/7/5	-0.029/0.011/0.049	increase	2.212	0.027		holds (sig increase)
B_SAMac_score	Macrophage	with_HL2	50	15	4stage	disease_only	MASH/Advanced	7/5	0.011/0.049	increase	1.543	0.1229		attenuates (trend, NS)
B_SAMac_score	Macrophage	with_HL2	50	15	binary	all	HC/MASH	3/12	-0.029/0.028	increase	1.588	0.1124		attenuates (trend, NS)
B_SAMac_score	Macrophage	with_HL2	50	15	binary	disease_only	MASH	12	0.028	flat	0	1		loses (non-monotonic/NS)
B_SAMac_score	Macrophage	noHL2	10	33	4stage	all	Healthy/MASLD/MASH/Advanced	2/4/19/8	-0.023/-0.021/0.011/0.042	increase	1.643	0.1004		attenuates (trend, NS)
B_SAMac_score	Macrophage	noHL2	10	33	4stage	disease_only	MASLD/MASH/Advanced	4/19/8	-0.021/0.011/0.042	increase	1.341	0.1799		loses (NS)
B_SAMac_score	Macrophage	noHL2	10	33	binary	all	HC/MASLD/MASH	2/4/27	-0.023/-0.021/0.011	increase	1.347	0.1781		loses (NS)
B_SAMac_score	Macrophage	noHL2	10	33	binary	disease_only	MASLD/MASH	4/27	-0.021/0.011	increase	0.943	0.3458		loses (NS)
B_SAMac_score	Macrophage	noHL2	20	24	4stage	all	Healthy/MASLD/MASH/Advanced	2/3/13/6	-0.023/-0.037/0.005/0.053	increase	2.336	0.0195		holds (sig increase)
B_SAMac_score	Macrophage	noHL2	20	24	4stage	disease_only	MASLD/MASH/Advanced	3/13/6	-0.037/0.005/0.053	increase	2.101	0.0356		holds (sig increase)
B_SAMac_score	Macrophage	noHL2	20	24	binary	all	HC/MASLD/MASH	2/3/19	-0.023/-0.037/0.022	increase	1.376	0.1689		loses (NS)
B_SAMac_score	Macrophage	noHL2	20	24	binary	disease_only	MASLD/MASH	3/19	-0.037/0.022	increase	0.909	0.3634		loses (NS)
B_SAMac_score	Macrophage	noHL2	30	18	4stage	all	Healthy/MASLD/MASH/	2/1/9/6	-	increa	1.7	0.07		attenuates

							Advanced		0.023/0.072/0.005/0.053	se	92	31		(trend, NS)
B_SAMac_score	Macrophage	noHL2	30	18	4stage	disease_only	MASLD/MASH/Advanced	1/9/6	0.072/0.005/0.053	decrease	1.191	0.2337		loses (NS)
B_SAMac_score	Macrophage	noHL2	30	18	binary	all	HC/MASLD/MASH	2/1/15	-0.023/0.072/0.022	increase	0.531	0.5957		loses (NS)
B_SAMac_score	Macrophage	noHL2	30	18	binary	disease_only	MASLD/MASH	1/15	0.072/0.022	decrease	-1.193	0.2328		loses (NS)
B_SAMac_score	Macrophage	noHL2	50	14	4stage	all	Healthy/MASH/Advanced	2/7/5	-0.023/0.011/0.049	increase	2.002	0.0453		holds (sig increase)
B_SAMac_score	Macrophage	noHL2	50	14	4stage	disease_only	MASH/Advanced	7/5	0.011/0.049	increase	1.543	0.1229		attenuates (trend, NS)
B_SAMac_score	Macrophage	noHL2	50	14	binary	all	HC/MASH	2/12	-0.023/0.028	increase	1.278	0.2012		loses (NS)
B_SAMac_score	Macrophage	noHL2	50	14	binary	disease_only	MASH	12	0.028	flat	0	1		loses (non-monotonic/NS)
C_NFkB_TLR_substate	Macrophage_SAMac	noHL2(by construction)	10	20	4stage	all	Healthy/MASLD/MASH/Advanced	2/1/11/6	0.000/0.000/0.000/0.551	increase	2.692	0.0071		holds (sig increase)
C_NFkB_TLR_substate	Macrophage_SAMac	noHL2(by construction)	10	20	disease_3level	disease_only	MASLD/MASH/Advanced	1/11/6	0.000/0.000/0.551	increase	2.673	0.0075	LOCKED z=2.67	holds (sig increase)
C_NFkB_TLR_substate	Macrophage_SAMac	noHL2(by construction)	10	20	binary_collapse_Adv	disease_only	MASLD/MASH	1/17	0.000/0.000	flat/dec	0.675	0.4999		loses (NS)

Table S24. Donor accounting (CONSORT-style) for the discovery cohort GSE202379.

block	row_id	analysis_or_step	cohort	compartment	inclusion_rule	n_Healthy	n_MASLD	n_MASH	n_Advanced	n_total	test	statistic	p_value	notes
A. Cohort funnel	A1	Donors profiled (whole discovery cohort)	GSE202379	All liver nuclei	No cell-count threshold	4	7	27	9	47				Discovery snRNA-seq; stage from Disease.status (Healthy/MASLD/MASH/Advanced)
A. Cohort funnel	A2	Macrophage nuclei retained after cell-type + purity QC	GSE202379	Macrophage	>=1 macrophage nucleus	4	7	25	9	45				2 donors retained no macrophage nuclei after purity filtering
A. Cohort funnel	A3	Macrophage donors passing >=20-nucleus threshold	GSE202379	Macrophage	>=20 macrophage nuclei	3	3	13	6	25				20 donors excluded (<20 macrophage nuclei); matches Table S6/S23
A. Cohort funnel	A4	Macrophage set after HL2 healthy-outlier removal	GSE202379	Macrophage	>=20 nuclei; HL2 removed	2	3	13	6	24				HL2 = pre-specified healthy-baseline outlier
A. Cohort funnel	A5	Macrophage disease-only set (PRIMARY)	GSE202379	Macrophage	>=20 nuclei; healthy excluded	0	3	13	6	22				Primary progression analysis MASLD->MASH->Advanced
A. Cohort	A6	HSC nuclei retained after cell-type +	GSE202379	HSC	>=1 HSC nucleus	4	7	27	9	47				

funnel		purity QC												
A. Cohort funnel	A7	HSC donors passing >=20-nucleus threshold	GSE202379	HSC	>=20 HSC nuclei	3	5	16	7	31				16 donors excluded (<20 HSC nuclei); matches Table S6
A. Cohort funnel	A8	HSC set after HL2 healthy-outlier removal	GSE202379	HSC	>=20 nuclei; HL2 removed	2	5	16	7	30				
A. Cohort funnel	A9	HSC disease-only set (PRIMARY)	GSE202379	HSC	>=20 nuclei; healthy excluded	0	5	16	7	28				
B. Per-analysis	B1	SAMac fraction stage-trend (PRIMARY)	GSE202379	Macrophage	>=20 nuclei; disease-only	0	3	13	6	22	Jonckheere-Terpstra	z=2.23	0.026	Table S23; Fig 2B panel displays the 4-stage distribution
B. Per-analysis	B2	SAMac fraction stage-trend (sensitivity: +healthy, HL2 removed)	GSE202379	Macrophage	>=20 nuclei; +2 healthy	2	3	13	6	24	Jonckheere-Terpstra	z=2.50	0.012	Table S23
B. Per-analysis	B3	SAMac fraction stage-trend (sensitivity: +healthy, HL2 retained)	GSE202379	Macrophage	>=20 nuclei; +3 healthy	3	3	13	6	25	Jonckheere-Terpstra	z=2.74	0.006	Value quoted in Fig 2B caption
B. Per-analysis	B4	SAMac signature-score stage-trend (PRIMARY)	GSE202379	Macrophage	>=20 nuclei; disease-only	0	3	13	6	22	Jonckheere-Terpstra	z=2.10	0.036	Table S23
B. Per-analysis	B5	SAMac signature-score stage-trend (sensitivity: +healthy, HL2 removed)	GSE202379	Macrophage	>=20 nuclei; +2 healthy	2	3	13	6	24	Jonckheere-Terpstra	z=2.34	0.020	Table S23
B. Per-analysis	B6	SAMac signature-score stage-trend (sensitivity: +healthy, HL2 retained)	GSE202379	Macrophage	>=20 nuclei; +3 healthy	3	3	13	6	25	Jonckheere-Terpstra	z=2.48	0.013	Value quoted in Fig 2G caption
B. Per-analysis	B7	NF-kB/TLR SAMac substate fraction stage-trend	GSE202379	Macrophage (SAMac)	>=10 SAMac nuclei; disease-only	0	1	11	6	18	Jonckheere-Terpstra	z=2.67	0.008	Table S23; Fig 2F. Substate-level threshold (>=10 SAMac nuclei)
B. Per-analysis	B8	HSC activation fraction stage-trend (discovery internal; NS)	GSE202379	HSC	>=20 nuclei; disease-only	0	5	16	7	28	Jonckheere-Terpstra	z=1.61	0.108	NS. 4-stage incl. healthy (n=31): z=0.72, p=0.47 (Fig 6D). Score metric disease-only: z=1.81, p=0.071
B. Per-analysis	B9	PDGF cross-donor ligand-receptor coupling (raw)	GSE202379	Macrophage + HSC	HSC>=20 & macrophage data; healthy incl.	3	4	16	7	30	Spearman	rho=0.54	0.002	PDGFC+ SAMac load vs HSC PDGFRB-downstream module
B. Per-analysis	B10	PDGF cross-donor coupling (stage-adjusted)	GSE202379	Macrophage + HSC	same 30 donors	3	4	16	7	30	partial Spearman	rho=0.49	0.006	Controls disease stage (partial correlation)

B. Per-analysis	B11	PDGFRB/receptor-hub sub-axis (stage-adjusted)	GSE202379	Macrophage + HSC	same 30 donors	3	4	16	7	30	partial Spearman	rho=0.46	0.011	Receptor-hub axis (incl. PDGFRB)
B. Per-analysis	B12	LIANA ligand-receptor inference (pooled)	GSE202379	SAMac + HSC substates	cells pooled across donors; disease-only					43	rank_aggregate (CellPhoneDB/NATMI/Connectome)	23,704 LR (11,698 mac<->HSC)	PDGFC->PDGFRA/B p<0.001	4,902 pooled cells across 43 disease donors; per-donor pervasiveness assessed separately (Table S12)
B. Per-analysis	B13	LIANA donor-pervasiveness (PDGFC->PDGFRB)	GSE202379	SAMac -> activated HSC	>=5 cells/side; disease-only					22	donor fraction expressing both	21/22 (95.5%)	-	>=10% source express ligand AND >=10% target express receptor (Table S12)
